# Risk-Aware Fault-Tolerant Multisensor Fusion for Human–Machine Decision Support in Autonomous Navigation and Mooring in Confined Waters

**DOI:** 10.3390/s26185702

**Published:** 2026-09-08

**Authors:** Sergey I. Kondratyev, Evgeniy V. Khekert, Nikita V. Martyushev, Boris V. Malozyomov, Vladislav V. Kukartsev, Valeriya V. Tynchenko, Tatyana Aleksandrovna Panfilova, Yadviga Aleksandrovna Tynchenko, Natalia I. Kozhukhova

**Affiliations:** 1 Rector’s Office, Admiral Ushakov Maritime State University, 93 Lenin Ave., Novorossiysk 353918, Russia; sikondr@gmail.com (S.I.K.); zur_mga@nsma.ru (E.V.K.); 2 Department of Information Technologies, Tomsk Polytechnic University, Tomsk 634050, Russia; 3 Department of Electrotechnical Complexes, Novosibirsk State Technical University, 20 Karl Marx Avenue, Novosibirsk 630073, Russia; borisnovel@mail.ru; 4 Department of Software Engineering, Siberian Federal University, Krasnoyarsk 660041, Russia; vlad_saa_2000@mail.ru (V.V.K.); vvtynchenko@sibsau.ru (V.V.T.); 5 Artificial Intelligence Technology Scientific and Education Center, Bauman Moscow State Technical University, Moscow 105005, Russia; 6 Department of Applied Computer Science, Russian State Agrarian University—Moscow Timiryazev Agricultural Academy, Moscow 127434, Russia; 7 Center for Continuing Education, Bauman Moscow State Technical University, Moscow 105005, Russia; t_pan80@mail.ru (T.A.P.); t080801@yandex.ru (Y.A.T.); 8 Department of Technological Machines and Equipment for the Oil and Gas Complex, Siberian Federal University, Krasnoyarsk 660041, Russia; 9 Department of Material Science and Material Technology, Belgorod State Technological University Named After V.G. Shukhov, Belgorod 308012, Russia; kozhuhovanata@yandex.ru

**Keywords:** heterogeneous sensor fusion, GNSS/INS integration, maritime radar, LiDAR, computer vision, navigation integrity, fault detection and isolation, autonomous mooring, human–machine decision support, confined waters

## Abstract

Autonomous navigation and mooring in confined waters require a navigation solution that remains reliable when individual sensing channels are delayed, unavailable, or environmentally degraded. A risk- and integrity-aware architecture was developed for joint processing of RTK-GNSS, inertial and heading measurements, short-range radar, LiDAR, camera, AIS, ultrasonic ranging, propulsion feedback, environmental, and mooring-line tension data. Asynchronous time alignment is combined with sensor-quality assessment, innovation-based fault detection and isolation, covariance adaptation, active-set reconfiguration, protection-level monitoring, and risk-dependent allocation of authority between automation and the operator. The system was evaluated during a five-day campaign comprising 40 runs, four operational phases, 480 synchronized evaluation epochs, and 16 controlled single-sensor or combined sensor-degradation events. Under nominal conditions, horizontal-position and heading RMSE were 0.043 m and 0.176°, respectively. All 64 fault-active diagnostic records were identified; mean event-log detection and controlled-recovery latencies were 3.17 s and 6.34 s. Horizontal protection-level coverage was 99.3% for single-fault epochs and 100% for combined-fault epochs. One combined-fault docking run contained five consecutive aborted decision epochs, while no unsafe-autonomy event was recorded. The measurements therefore indicate bounded degradation of the navigation solution and conservative transfer of authority when sensing integrity decreases. Because all injected-fault runs were acquired under adverse weather whereas nominal runs were acquired under calm or moderate conditions, the condition-class RMSE differences reported below are descriptive and must not be interpreted as isolated causal effects of sensor faults.

## 1. Introduction

Confined-water navigation and berthing impose a sensing problem that is qualitatively different from open-water route following. The vessel must maintain a reliable earth-referenced state while simultaneously estimating its relative pose with respect to channel boundaries, berth structures, nearby vessels, and mooring constraints. The admissible spatial margins contract as the operation progresses from restricted-channel transit to berth approach and final docking, whereas the consequences of a delayed or misleading estimate increase. Recent autonomous-berthing studies have therefore moved from single-source navigation toward combinations of RTK-GNSS, inertial sensing, LiDAR, radar, and vision, with the objective of obtaining both global state and local geometric observables [1,2,3,4].

No individual sensor modality is sufficient over the complete operational envelope. GNSS provides an absolute position and timing reference but is vulnerable to multipath, masking, correction-link interruption, and receiver-quality degradation near port infrastructure. Inertial sensors preserve short-term continuity but accumulate bias-driven drift, especially during the low-dynamic maneuvers typical of approach and docking. LiDAR offers high-resolution local geometry, yet its return density and effective range depend on precipitation, spray, reflectivity, and partial occlusion; camera measurements are affected by illumination, glare, shadows, and feature scarcity. Marine radar is comparatively robust to lighting but exhibits clutter, multipath, and limited geometric resolution at short range. AIS supports identity-aware cooperative perception but is asynchronous, delayed, and unavailable for non-cooperative targets. Radar–AIS–camera integration, robust AIS–visual association, and simulation-based association studies confirm both the complementarity of these channels and the need to retain modality-specific uncertainty [5,6,7].

The 24-channel arrangement used in this study should be interpreted as an experimental reference architecture rather than a mandatory production bill of materials. Existing maritime implementations already use narrower combinations such as RTK/IMU/LiDAR berthing perception [1], multi-LiDAR/MMW-radar fusion [2], 3D-LiDAR berthing assistance [3,4], and radar–AIS–camera perception [5,6]. A deployable system can therefore reuse sensors already carried by the vessel (GNSS/INS, gyrocompass, radar, and AIS) and add only the phase-critical exteroceptive, proximity, and line-load channels required by the intended autonomy function. The complete suite was retained here to provide heterogeneous redundancy and to evaluate reconfiguration and common-cause behavior under controlled degradations; no claim is made that all 24 channels constitute the minimum-cost production configuration.

The central engineering issue is therefore not only multisensor accuracy but also the credibility of the fused solution. A filter can remain numerically stable while producing hazardously misleading information if the assumed measurement covariance does not reflect the current sensing environment or if a persistent fault is absorbed into the state estimate. Robust and adaptive GNSS/INS estimators address part of this problem by modifying measurement weights in response to residuals or satellite geometry, while fault-tolerant navigation filters add diagnostic reallocation of sensor information [8,9,10]. For safety-related autonomy, however, a point estimate and covariance are insufficient unless they are connected to explicit alert limits, protection levels, continuity requirements, and the probability that the actual error exceeds the declared bound [11,12].

Maritime integrity terminology in this study follows the IMO-oriented interpretation summarized by Zalewski and co-workers [13,14]: integrity is the ability to provide a timely warning when the navigation solution should not be used, continuity concerns maintaining the required function over the relevant operation interval, availability is the fraction of time the function is usable, and the alert limit is the maximum admissible error before an alarm is required. Accordingly, protection-level non-envelopment, alert-limit exceedance, continuity loss, and an HMI-event are reported as distinct outcomes rather than treated as synonyms.

Fault-tolerant fusion therefore requires a coordinated sequence of functions: data-quality assessment, fault detection, fault isolation, covariance adaptation, sensor exclusion or down-weighting, controlled re-entry, and integrity monitoring. Innovation statistics and quality factors can redistribute information among navigation subsystems, but a maritime application is broader because the active sensor set changes with operational phase. AIS and radar dominate traffic awareness during channel transit; LiDAR, cameras, and short-range ranging become progressively more important during berth approach; propulsion, proximity, and line-tension channels become safety-critical during final docking and line securing. A fixed fusion topology is therefore structurally mismatched to the task.

The uncertainty of the fused state must also be propagated into the decision layer. When navigation risk is high but integrity is strong, automatic protective action may be justified. When risk appears low but the integrity bound is unreliable, continued full autonomy can be unsafe because the apparent margin may be an artefact of degraded sensing. Human–machine cooperation and supervisory shared-control studies indicate that the operator’s role evolves from continuous manual control toward supervision, confirmation, and takeover under exceptional conditions [11,12]. Maritime positioning research likewise distinguishes accuracy from integrity and emphasizes application-specific alert limits and performance standards [13,14].

The methodological basis combines multisensor-fusion theory, reliability analysis for intelligent technical systems, and fault detection, isolation, and reconfiguration [15,16,17,18]. Shared-control research treats authority as a continuously allocated system variable rather than a fixed manual/automatic choice [19,20]. Maritime safety studies further show that remote supervision and degraded operating states change the relevant failure pathways for autonomous vessels [21,22].

The objective of this study is to develop and experimentally evaluate an end-to-end architecture for the continuous operational sequence comprising restricted-channel transit, berth approach, final docking, and mooring-line securing. The contributions are as follows: (1) a phase-adaptive heterogeneous sensing architecture combining global navigation, external perception, near-field ranging, actuation, environmental, and line-load channels; (2) an asynchronous quality-aware fusion formulation in which effective measurement covariance depends on availability, latency, innovation consistency, environmental suitability, and cross-sensor agreement; (3) a fault-detection, isolation, reconfiguration, and controlled-recovery mechanism; (4) phase-dependent position, heading, relative-pose, and line-load integrity monitoring; (5) propagation of state and integrity uncertainty into operational risk; and (6) a joint risk–integrity policy for authority allocation. The experimental logic follows the principle that multisensor conclusions must be connected to independently registered measurements and field-level operating conditions [23], while the safety argument is organized as a traceable system-theoretic chain rather than as a collection of isolated algorithms [24]. Section 2 reviews the relevant literature; Section 3 and Section 4 formulate the architecture and algorithms; Section 5 describes the platform and protocol; Section 6 reports the results; and Section 7, Section 8 and Section 9 provide discussion, limitations, and conclusions.

Throughout the manuscript, “confined waters” is the umbrella term for the operational environment. “Restricted-channel transit” denotes one specific experimental phase within confined waters, whereas “littoral” is retained only when describing the terminology or scope of cited prior studies.

## 2. Related Work and Research Gap

### 2.1. Multisensor Positioning and Environmental Perception in Maritime Operations

GNSS/INS integration remains the principal source of global vessel position, velocity, attitude, and time continuity. In confined-water nearshore operations, however, the navigation state must be combined with heterogeneous target tracking and nearshore mapping; experimental studies have demonstrated radar-, camera-, AIS-, and LiDAR-based tracking and static mapping under shoreline clutter and restricted visibility [25,26]. Wu et al. combined radar, AIS, and camera information for integrated ship-motion perception in inland waterways [5], while Qu et al. addressed the mismatch between identity-rich but delayed AIS reports and appearance-rich visual observations [6]. Data association remains a separate uncertainty source and must be validated against dynamic scenarios and sensor-specific observation models [7].

Berthing perception imposes tighter geometric requirements. Robust LiDAR tracking and camera-fusion studies have extended maritime perception toward congested and dynamically occluded environments [27,28]. Recent survey work shows that maritime datasets and algorithms now cover detection, tracking, semantic perception, multimodal fusion, and SLAM, while cross-domain laser–vision reviews confirm that fusion performance depends strongly on calibration, representation, and failure-aware weighting [29,30]. Multi-LiDAR/radar fusion can estimate berth distance, approach angle, velocity, and yaw [2], while shipborne 3D-LiDAR methods provide complementary berth-state observables [3,4]; the present study extends this line by treating local perception quality as a time-varying state that directly modifies covariance, fault status, integrity bounds, and decision authority.

### 2.2. Sensor Quality Assessment, Fault Detection, and Navigation Integrity

Accuracy and integrity are related but non-equivalent properties. Accuracy characterizes the realized estimation error, whereas integrity concerns the ability to provide a timely and sufficiently conservative bound on that error. Nonlinear state-estimation theory provides the mathematical basis for propagating means and covariances through nonlinear sensor models [31], while adaptive moving-base RTK filtering shows how ambiguity validation can modify stochastic weighting in real time [32]. State-domain autonomous integrity monitoring addresses slowly growing faults that may evade instantaneous residual tests [33], and robust stochastic modelling improves RTK behavior when nominal noise assumptions fail [34]. GNSS/INS integration with multi-element antennas further illustrates how sensing architecture changes both observability and fault exposure [35]. These results complement the robust state/protection-level and robust-adaptive filtering approaches used as direct methodological precedents in this study [8,9]. For heterogeneous fusion, the diagnostic layer must distinguish transient noise growth from persistent bias, drift, delay, dropout, occlusion, saturation, and cross-modal inconsistency. Innovation-based chi-square testing is computationally attractive but can be unreliable when model error and sensor faults have similar residual signatures. Persistent-window tests, cross-sensor residuals, availability and latency flags, environmental suitability, and controlled recovery conditions are therefore combined here. Fuzzy quality factors within fault-tolerant navigation provide a relevant precedent [10]; the present architecture extends the diagnostic output into phase-specific protection levels, risk propagation, and authority allocation.

### 2.3. Human–Machine Decision Support for Autonomous Navigation and Mooring

Human–machine cooperation in maritime autonomy includes situation awareness, supervisory control, cooperative decision making, and transfer of authority. Reviews and shared-control studies indicate that autonomous systems, remote operators, and onboard personnel retain complementary capabilities in uncertain or exceptional conditions [11,12]. Remote-control-center research treats situation awareness as a distinct design problem rather than a simple relocation of the bridge [36]. System-theoretic analyses emphasize feedback, control structure, and unsafe interactions [37], whereas human-factor studies identify communication, workload, competence, and interface design as persistent safety factors [38]. In this experiment, operator-event records are used only to evaluate authority transitions, response timing, and executed actions. Workload, trust, usability, and general operator behavior were outside the experimental protocol.

### 2.4. Research Gap and Positioning of the Present Study

Published work commonly treats global navigation, local berthing perception, fault management, integrity monitoring, and authority allocation as separate subsystems. Global and local estimators are often validated independently; fault-management studies frequently report accuracy loss without a calibrated protection level; and authority transitions are usually driven by geometric risk or predefined automation levels without explicit use of measurement integrity. The architecture considered here links sensor quality, fault mode, effective covariance, fused-state protection levels, phase-specific hazard probabilities, and authority state in one traceable chain. Its primary contribution is therefore located at the interfaces among sensing, estimation, assurance, and supervisory decision support. Table 1 compares these interfaces across representative approaches and distinguishes sensor-domain breadth from assurance depth.

Table 1 is intended as a scope comparison rather than a ranking of prior methods. The cited studies address different scientific questions, and the absence of a component in the table should not be interpreted as a deficiency when that component lies outside the original study scope. In the present work, the error-state EKF, normalized-innovation testing, covariance inflation, Joseph-form covariance update, sequential asynchronous measurement updates, persistence counters, hysteresis, and rule-based authority modes are standard or established techniques. The methodological contribution is their phase-dependent integration into one auditable chain in which sensor quality and FDI state modify effective covariance, protection levels, operational risk, and authority allocation, together with experimental evaluation of that complete chain in a controlled confined-water campaign. The novelty claim is therefore system-level integration and experimental coupling, not invention of each individual estimator or diagnostic primitive.

## 3. System Architecture and Problem Formulation

### 3.1. Operational Phases and Confined-Water Scenario

**Figure 1:**
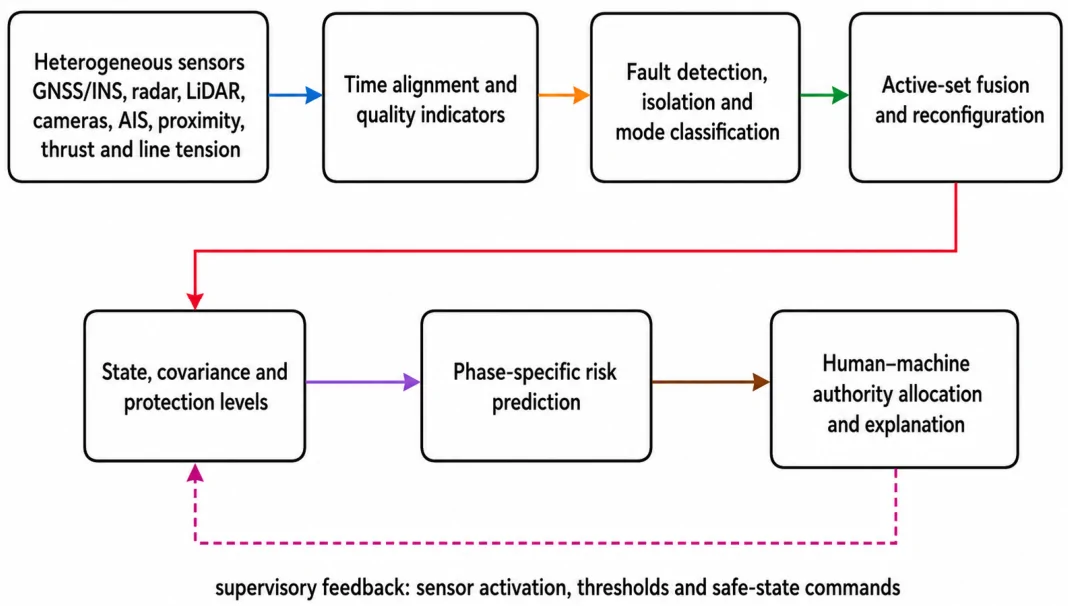
End-to-end architecture of the risk- and integrity-aware multisensor decision-support framework.

The operational sequence is divided into four phases because the observable state, dominant hazards, and admissible error bounds change materially over time. During restricted-channel transit, the primary objectives are earth-referenced position, heading, velocity, channel-boundary clearance, and traffic-relative state. The navigation core is supported by AIS and radar, while the horizontal and heading alert limits used in the experiment are 2.0 m and 5.0°, respectively. During berth approach, the state is augmented by berth-relative distance, angle, and closing speed; radar, LiDAR, cameras, and short-range ranging are activated with increasing weight. Final docking uses a 0.5 m relative-position alert limit and a 3.0° relative-heading alert limit, with low-speed actuation and proximity observables dominating the decision. During line securing, the safety state is extended to individual line tension, thrust response, wind/current disturbance, and overload margin. The four phases are represented by ten experimental runs each. In every phase, six runs were conducted under nominal sensing conditions, three included a controlled single-sensor degradation, and one included a combined degradation. This balanced phase structure permits the same fault-management and authority logic to be evaluated under different sensor-relevance patterns. Because individual fault mechanisms have fewer repetitions than the aggregate phase classes, fault-specific estimates are interpreted for the recorded experimental conditions rather than as universal equipment failure rates. The functional sequence that connects heterogeneous sensing to the human–machine decision layer is summarized in Figure 1. In Figure 1, the upper row represents forward information processing, whereas the lower row represents safety interpretation and authority output. The return paths are equally important because they allow diagnosed degradation and safety state to modify sensor admission, thresholds, and fail-safe commands.

Figure 1 emphasizes that fault tolerance is implemented as a closed supervisory loop rather than as a single filtering operation. Sensor activation, covariance adaptation, integrity monitoring, and authority allocation remain mutually coupled throughout the maneuver. The diagram also distinguishes estimation from assurance: the fused posterior is not passed directly to control, but first acquires protection-level and phase-specific risk semantics. This separation is the structural basis for preventing a numerically available but insufficiently credible solution from retaining unrestricted autonomy.

### 3.2. Heterogeneous Sensor Configuration

The experimental inventory contains 24 documented channels. The navigation core comprises a Septentrio AsteRx-U3 RTK-GNSS (Septentrio N.V., Leuven, Belgium) receiver at 20 Hz, a VectorNav VN-300 IMU (VectorNav Technologies, LLC, Dallas, TX, USA) at 100 Hz, and a KVH P-1775 gyrocompass (KVH Industries, Inc., Bristol, RI, USA) at 100 Hz. External perception includes a Navtech CTS350-X short-range radar (Navtech Radar Ltd., Ardington, Wantage, UK) and an Ouster OS1-64 3D LiDAR (Ouster, Inc., San Francisco, CA, USA) at 10 Hz, two Basler ace 2 cameras (Basler AG, Ahrensburg, Germany) at 20 Hz, and an SRT Marine Class A AIS receiver (SRT Marine Systems plc, Midsomer Norton, UK) at 1 Hz. Four Pepperl + Fuchs UC6000 ultrasonic sensors (Pepperl+Fuchs SE, Mannheim, Germany) provide near-field ranging. Propulsion and steering feedback are measured by two TC-100 thrust channels, a Novotechnik RFC-4800 rudder sensor (Novotechnik Messwertaufnehmer OHG, Ostfildern, Germany), and two HBM T40B shaft-speed channels (Hottinger Brüel & Kjær GmbH, Darmstadt, Germany). Four HBM U10M load cells (Hottinger Brüel & Kjær GmbH, Darmstadt, Germany) measure mooring-line tension, while Gill WindSonic (Gill Instruments Ltd., Lymington, Hampshire, UK) and Nortek Aquadopp instruments (Nortek AS, Rud, Norway) characterize wind and current. A software-derived fused-object track completes the 24-channel architecture. All channels are referenced to an east–north–up earth frame and a body frame defined by x forward, y starboard, and z down. The acquisition configuration distinguishes high-rate (100 Hz), medium-rate (10–20 Hz), and low-rate (1 Hz) streams. Twelve synchronized evaluation epochs were retained from each run for the common statistical dataset. Manufacturer, model, nominal accuracy, rate, timestamp source, latency, mounting coordinates, and calibration metadata are documented in the experimental inventory; only equipment serial numbers and the exact operational-site identifier are anonymized.

Table 2 maps each sensor group to its measured observables, native update rate, and operational role. The table is intended not merely as an equipment inventory, but as an observability map that explains which channel groups constrain global navigation, local berth geometry, actuator response, environmental disturbance, and mooring loads during each phase.

Table 2 demonstrates that the sensor suite is phase complementary rather than merely redundant. Navigation-core channels provide continuity, external-perception channels define surrounding geometry and traffic, and the near-field and line-load channels become dominant as the vessel approaches the berth. The table also exposes an important asymmetry in redundancy. Global position and relative geometry are supported by heterogeneous physical principles, whereas line tension is measured by multiple channels of the same sensor class; this difference anticipates the higher sensitivity of line-load estimation observed in Section 6.

**Figure 2:**
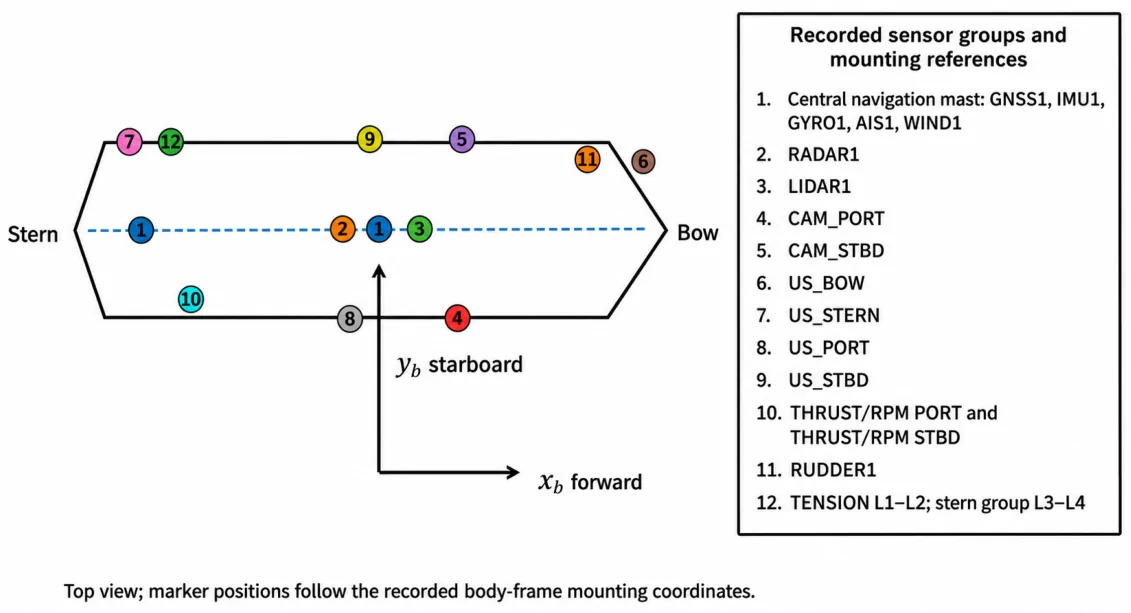
Sensor placement and body-frame coordinates on the experimental vessel; numbered markers correspond to the recorded sensor groups. Group 12 comprises four separate line-tension load-cell channels: L1–L2 at the forward/bow mooring station and L3–L4 at the aft/stern station.

The recorded mounting coordinates and functional distribution of the 24 sensor channels are presented in Figure 2. The placement view is required because lever arms, fields of view, occlusion patterns, and the physical separation of proximity and line-load measurements influence both observability and common-cause vulnerability.

Figure 2 shows that the architecture combines centrally mounted navigation sensors with distributed near-field, propulsion, and line-load channels. This spatial diversity is important because global position, berth-relative geometry, actuator response, and mooring loads cannot be observed reliably from one mounting location. Central placement minimizes lever-arm uncertainty for the navigation core, whereas distributed edge and line measurements improve local geometric and load observability. At the same time, this distribution makes temporal and extrinsic calibration indispensable: spatial diversity is beneficial only when all observations are transformed to a common epoch and body frame. In Figure 2, the four group-12 load cells are grouped under one functional label for readability; they are not co-located, and their recorded body-frame mounting coordinates remain distinct.

### 3.3. State Vector, Coordinate Frames, and Dynamic Model

The estimator uses four coordinate systems. The navigation frame n is a local east–north-up frame fixed to the experimental area; the body frame b has its x-axis directed forward, y-axis to starboard, and z-axis downward; each sensor frame $s_{i}$ is related to b by a calibrated rigid transformation; and the berth frame B is fixed to the surveyed berth geometry. The state is organized as a persistent navigation core and phase-dependent operational extensions. This separation avoids carrying weakly observable docking or line-load states during channel transit while preserving a common covariance representation across phase transitions. Joint temporal/spatial calibration and visual–inertial self-calibration provide established procedures for maintaining a coherent set of sensor-frame transformations and time offsets [39,40].

(1) $$x_{k}={\left[{\left(p_{k}^{n}\right)}^{T}\;\;\;{\left(v_{k}^{n}\right)}^{T}\;\;\;\eta_{k}^{T}\;\;\;b_{a,k}^{T}\;\;\;b_{g,k}^{T}\;\;\;{\left(x_{k}^{rel}\right)}^{T}\;\;\;{\left(x_{k}^{env}\right)}^{T}\;\;\;\tau_{k}^{T}\right]}^{T},$$

The implementation uses a 15-state persistent navigation core: three position states, three velocity states, three attitude states, three accelerometer-bias states, and three gyroscope-bias states. The berth-relative extension adds five states (two-dimensional relative position, two-dimensional closing velocity, and relative heading), the horizontal environmental-disturbance extension adds four states (two wind and two current components), and line securing adds four line-tension states. Thus, the maximum state-vector dimension is $n_{x}=28$. The active phase-dependent subset is $n_{x}=19$ during berth approach, $n_{x}=19$ during final docking, and $n_{x}=23$ during line securing when the berth-relative and four line-load states are active. The 15-state core remains active in transit. In Equation (1), $x_{k}$ denotes the complete estimator state vector; $p_{k}^{n}$ and $v_{k}^{n}$ are position and velocity in the navigation frame; $\eta_{k}$ is the attitude-state vector; $b_{a,k}$ and $b_{g,k}$ are the accelerometer- and gyroscope-bias vectors; $x_{k}^{rel}$ is the berth-relative state extension; $x_{k}^{env}$ is the environmental-disturbance state extension; and $\tau_{k}$ is the mooring-line tension-state vector.

The navigation core is propagated with a strapdown inertial model. At the low vessel speeds of the experiment, earth-rotation and transport-rate terms are retained in the implementation but are omitted from the compact presentation below because their contribution over one update interval is substantially smaller than sensor-bias and environmental-disturbance terms. The continuous-time nominal dynamics are

(2) $${\dot{x}}_{nav}={\left[{\left(v^{n}\right)}^{T},{\left(C_{b}^{n}\left(f_{m}-b_{a}-n_{a}\right)+g^{n}+d^{n}\right)}^{T},{\left(T\left(\eta \right)\left(\omega_{m}-b_{g}-n_{g}\right)\right)}^{T},{n_{b_{a}}}^{T},{n_{b_{g}}}^{T}\right]}^{T},$$

where $C_{b}^{n}$ is the direction-cosine matrix from the body to the navigation frame; $f_{m}$ and $\omega_{m}$ are the specific-force and angular-rate measurements; $n_{a}$ and $n_{g}$ are the corresponding white measurement-noise processes; $g^{n}$ is the gravity vector; $d^{n}$ is the estimated acceleration contribution of wind, current, and unmodelled low-speed hydrodynamic effects; T(η) maps body angular rate into Euler-angle rate; and $n_{b_{a}}$ and $n_{b_{g}}$ drive the random-walk bias models. The berth-relative states are propagated from the transformed global vessel state and the surveyed berth pose, while line tensions are treated as slowly varying states driven by measured load-cell increments and process noise.

For stochastic propagation, the accelerometer and gyroscope driving terms are modelled as zero-mean, axis-wise Gaussian white processes over each propagation interval, whereas the accelerometer- and gyroscope-bias states follow independent random walks. The associated continuous-time noise intensities are established from pre-run calibration and stable nominal segments and are held fixed within an operational phase. Only the disturbance, berth-relative, and line-load process-noise blocks are phase adapted in the continuous spectral-density model. The exact campaign-specific inertial white-noise densities and bias random-walk intensities are part of the processing-configuration material rather than the synchronized reporting export and are therefore not reconstructed retrospectively from the analysis table.

The error-state formulation follows robust tightly coupled navigation practice [8,9]. Between measurement arrivals, the nonlinear nominal state is propagated with the inertial and actuation inputs, whereas the covariance is propagated through the linearized error dynamics. The modular decomposition also follows system-level modelling practice in which interacting subsystems retain explicit interfaces and uncertainty states [41].

(3) $$x_{k+1}^{-}=f(x_{k}^{+},u_{k},\Delta t_{k}),\;\Phi_{k}=\exp(F_{k}\Delta t_{k}),$$

where $x_{k+1}^{-}$ is the predicted state; $x_{k}^{+}$ is the posterior state estimate; $u_{k}$ collects the inertial, thrust, shaft-speed, and rudder inputs available over the interval; $\Delta t_{k}$ is the actual elapsed time; *f()* is the nonlinear propagation operator; $\Phi_{k}$ is the discrete state-transition matrix; and $F_{k}$ is the continuous-time Jacobian of the error dynamics evaluated at the posterior state.

(4) $$P_{k+1}^{-}=\Phi_{k}P_{k}^{+{\Phi_{k}}^{T}}+Q_{k},Q_{k}={\int \Phi \left(\tau \right)}_{0}^{\Delta t_{k}}GQ_{c}G^{T}\Phi^{T}\left(\tau \right)d\tau ,$$

where $P_{k}^{+}$ and $P_{k+1}^{-}$ are the posterior and predicted error covariances; *G* maps the continuous driving noises into the error state; $Q_{c}$ is the continuous spectral-density matrix; and $Q_{k}$ is the corresponding discrete process-noise covariance. Process-noise levels are phase dependent: disturbance and relative-pose uncertainty are increased during berth approach, while line-tension process noise is activated during line securing. This phase dependence prevents overconfident propagation when environmental forcing or contact geometry dominates the state evolution. Environmental-process uncertainty is treated as a time-varying stochastic input rather than a fixed nuisance constant, consistent with predictive modelling of weather-dependent technical-system states [42].

### 3.4. Asynchronous Measurement Model and Time Alignment

The sensor system is asynchronous by construction: IMU and gyrocompass channels operate at 100 Hz, RTK-GNSS and cameras at 20 Hz, radar and LiDAR at 10 Hz, and AIS and environmental channels at 1 Hz. Each observation is therefore processed at its corrected acquisition time rather than being forced onto a common raw-data grid. For sensor i and its local sample index l, the generic measurement equation is

(5) $$z_{i,l}=h_{i}\;\left(x({\tilde{t}}_{i,l})\right)+v_{i,l},\;v_{i,l}\sim N(0,R_{i,0}),$$

where $z_{i,l}$ is the observation vector; $h_{i}\;\left(\cdot \right)$ is the nonlinear sensor-specific measurement operator; $x({\tilde{t}}_{i,l})$ is the vessel state at the corrected observation time; $v_{i,l}$ is zero-mean measurement noise; and $R_{i,0}$ is the nominal covariance obtained from calibration and stable nominal segments. The measurement operators include absolute position and velocity for RTK-GNSS, specific force and angular rate for the IMU, heading for the gyrocompass, range-bearing or relative Cartesian coordinates for radar and LiDAR, visual feature or berth-pose observations for the cameras, cooperative target states for AIS, near-field range for ultrasonic sensors, actuation feedback for the propulsion channels, environmental forcing, and line tension.

For the camera and LiDAR channels, the estimator does not fuse raw pixels or individual point-cloud samples directly. A modality-specific perception front end first extracts and associates berth edges, obstacle or target features, relative-pose or range-bearing primitives, together with confidence/support metadata. Consequently, an observation may be absent when no valid feature is recognized, and its effective uncertainty can vary substantially with scene geometry, illumination, precipitation, spray, occlusion, reflectivity, segmentation/tracking quality, and data-association ambiguity. A missed feature produces no measurement update rather than a numerical zero; detected feature products are passed to the fusion layer with their current quality/confidence and cross-sensor consistency. This scenario dependence is why camera/LiDAR environmental suitability and innovation/association consistency enter the trust representation explicitly instead of being absorbed into one fixed sensor-noise value.

Timestamp handling explicitly separates the device timestamp from the estimated communication and processing delay.

(6) $$t_{i,l}=t_{i,l}^{stamp}-{\hat{\delta }}_{i,l},\;\Delta t_{i,l}=t_{i,l}-t_{k}^{+},$$

where $t_{i,l}^{stamp}$ is the received device timestamp; ${\hat{\delta }}_{i,l}$ is the estimated channel delay, including any calibrated clock offset; tilde $t_{i,l}$ is the corrected measurement time; $t_{k}^{+}$ is the time of the most recent posterior state; and $\Delta t_{i,l}$ is the propagation interval required before assimilating the observation. Delay estimates and residual time offsets are retained in the data-quality record and directly influence the trust score.

Measurements arriving after the estimator has advanced beyond their corrected time are treated as out-of-sequence observations when they remain within the retained state buffer.

(7) $$\begin{array}{c} \left({\hat{x}}^{+}\left(t\right),P^{+}\left(t\right)\right)=U_{i}\left[P\left({{\hat{x}}_{k}}^{+},{P_{k}}^{+},\Delta t_{i,l}\right),z_{i,l}\right],\\ \left({{\hat{x}}_{kc}}^{+},{P_{kc}}^{+}\right)=P\left({\hat{x}}^{+}\left(t\right),P^{+}\left(t\right),t_{kc}-t\right) \end{array}$$

where *P()* is the propagation operator; *U_i_()* is the update associated with sensor *i*; *t_kc_* is the current estimator time; and ${\hat{x}}^{+}(\tilde{t}),P^{+}(\tilde{t})$ are the delayed posterior state and covariance. After the delayed update, the stored inputs and accepted measurements are replayed to the current time. Observations older than the buffer horizon or with an excessive absolute time offset are not fused; they remain available for diagnostics and post-processing. This approach preserves the native measurement timing and avoids creating artificial correlations through blanket interpolation. Buffering and replay are also consistent with robust-perception architectures in which temporal alignment and state-history management are integral parts of the estimator rather than preprocessing conveniences [43]. In Equation (7), t denotes the corrected observation time defined by Equation (6); the second propagation returns the delayed posterior to the current estimator time.

**Figure 3:**
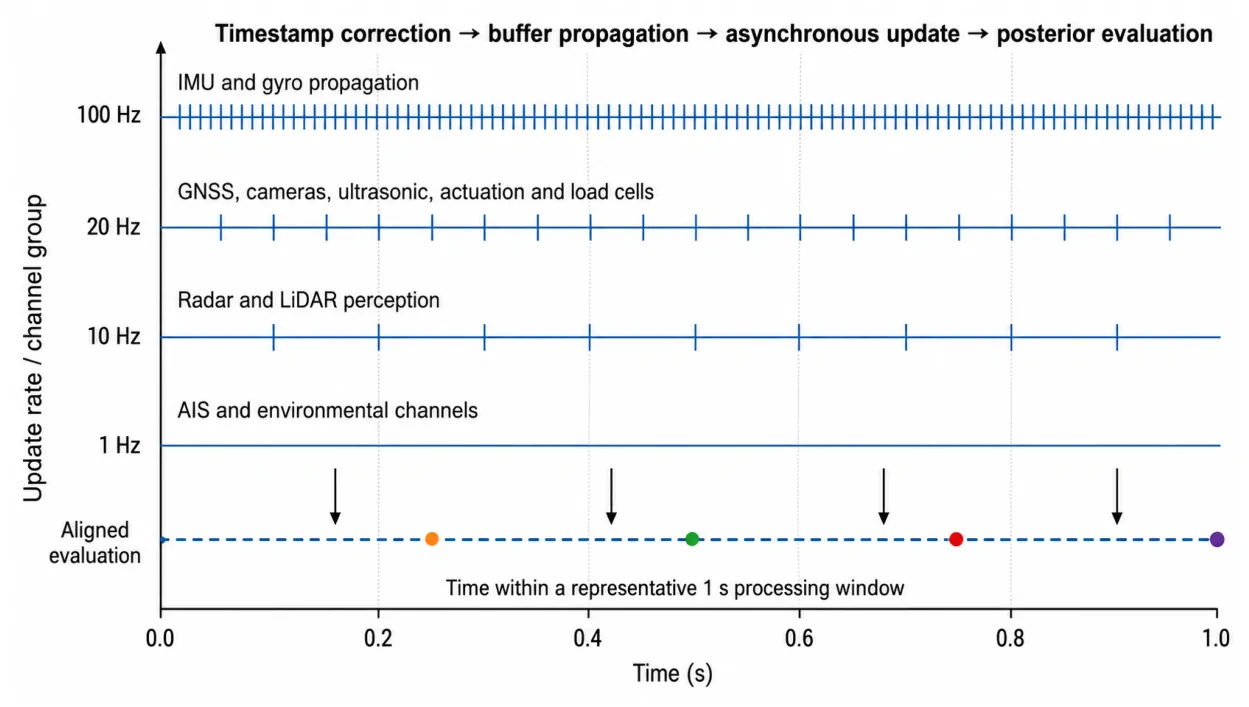
Multi-rate acquisition, timestamp alignment, asynchronous updating, and synchronized evaluation workflow.

Figure 3 summarizes the multi-rate workflow and clarifies the relation between native acquisition and statistical evaluation. The 480 synchronized epochs are posterior comparison points derived after event-driven estimation; they are not the fusion rate. The rows in Figure 3 represent the 100 Hz propagation stream, the 20 Hz measurement group, the 10 Hz perception group, and the 1 Hz cooperative/environmental group, while the bottom row marks aligned evaluation epochs.

Figure 3 distinguishes native acquisition rate from the synchronized epochs used for statistical comparison. High-rate inertial propagation is retained between slower exteroceptive updates, while timestamp correction and buffering prevent the evaluation dataset from being interpreted as the estimator’s native operating frequency. This distinction is essential for interpreting the reported detection and recovery latencies: those values belong to the synchronized diagnostic log, whereas the estimator processes native measurements between evaluation epochs. The figure therefore prevents the experimental reporting grid from being mistaken for the online update schedule. More precisely, the system has two distinct clocks: an asynchronous event clock at which each admissible sensor observation updates the posterior at its corrected timestamp, and a reporting clock at which selected posterior states are sampled at common epochs for statistical comparison. The aligned markers therefore do not define a periodic fusion clock or an estimator output rate.

This timing distinction also motivates the quality layer that follows. Because asynchronous observations arrive with different and time-varying reliability, each accepted sensor event is assigned its own quality components before it is allowed to modify covariance, sensor admission, and ultimately the fused posterior.

### 3.5. Sensor-Quality Vector and Trust Representation

A quality vector is constructed for every channel and accepted observation. Equation (8) contains exactly six normalized components in [0, 1]: availability, latency/time-offset quality, noise quality, innovation/cross-sensor consistency, environmental suitability, and recovery/re-entry status. The raw device quality flag is retained as source metadata and as a hard plausibility/admission gate; it is not a seventh numerical component of $q_{i,k}$. This distinction removes the apparent six-versus-seven discrepancy.

(8) $$q_{i,k}={\left[a_{i,k}\;\;l_{i,k}\;\;n_{i,k}\;\;c_{i,k}\;\;e_{i,k}\;\;r_{i,k}\right]}^{T},\;0\leq q_{i,k}^{\left(j\right)}\leq 1,$$

where the first two terms are the availability and latency scores; $n_{i,k}$ is the noise-quality score; $c_{i,k}$ is the innovation and cross-sensor consistency score; the remaining terms are the environmental-suitability and recovery/re-entry scores. Availability is binary before smoothing, whereas the other components are continuous. For example, environmental suitability for LiDAR and camera channels decreases with precipitation, spray, glare, or occlusion; AIS latency quality decreases with message age; and ultrasonic quality decreases in the presence of outliers, blind-zone returns, or inconsistent neighbouring ranges. Explicitly, $a_{i,k}$, $l_{i,k}$, $e_{i,k}$, and $r_{i,k}$ denote the availability, latency/time-offset, environmental-suitability, and recovery/re-entry scores, respectively.

Normalization is performed separately for each quality component of each sensor observation; it is not a normalization of the fused state vector or of individual navigation-state components. Each raw indicator is mapped monotonically to [0, 1] using fixed sensor- or modality-specific lower and upper reference limits established before online operation from calibration, manufacturer specifications and device constraints, protocol limits, and stable nominal segments. The corresponding higher-is-better or lower-is-better bounded mapping is then applied, while availability remains binary before smoothing. These normalization limits are held fixed during a run and are not inferred from the evolving fused state or from reference-state/ground-truth error.

The components are converted into a bounded trust coefficient using a sensor-specific calibrated mapping.

(9) $$\gamma_{i,k}=\sigma \;\left(\beta_{i,0}+\beta_{i}^{T}q_{i,k}-\beta_{D,i}D_{i,k}^{2}\right),\;\sigma (s)=\frac{1}{1+e^{-s}},$$

where $\gamma_{i,k}$ is the trust coefficient; σ () is the logistic function; $\beta_{i,0}$ and $\beta_{i}$ are calibration coefficients; $\beta_{D,i}$ controls the penalty associated with the exponentially averaged normalized innovation statistic $D_{i,k}^{2}$. The mapping is monotone with respect to each quality component and is calibrated on nominal and labelled-degradation intervals without using the reference-state error as an online input. This restriction prevents circular validation: ground truth is used to evaluate the estimator, not to generate operational trust.

Trust is interpreted as an estimator-control variable rather than a probability of correctness. Values near one retain the nominal covariance, intermediate values trigger covariance inflation and closer diagnostic supervision, and values below the modality-specific admission threshold lead to temporary exclusion. Re-entry requires both an acceptable innovation sequence and a recovery score above its threshold, thereby preventing a failed channel from immediately regaining full influence after a single plausible sample.

The calibration values and initial settings for the normalized quality vector in Equation (9) are presented next. For RTK-GNSS, IMU/gyro, radar, LiDAR, camera, AIS, ultrasonic, actuation-feedback, mooring-line tension, and environmental channels, the same initial confidence-map parameterization is used: $\beta_{i,0}=-4.5$, $\beta_{i}=[1.5,1.5,1.5,1.5,1.5,1.5]$, and $\beta_{D,i}=0.50$. This places the logistic midpoint close to an average normalized quality of 0.50 while retaining a strong penalty for persistent normalized innovation. The initial admission threshold is $\gamma_{i}^{adm}=0.50$; the covariance-scale limits in Equation (10) are $\alpha_{i}^{min}=1.0$ and $\alpha_{i}^{max}=6.5$; and the Equation (11) initial smoothing/rate-limit settings are $\lambda_{i}=0.80$, $\Delta_{i}^{-}=0.25$, and $\Delta_{i}^{+}=1.00$ per accepted observation.

### 3.6. Research Hypotheses and Evaluation Logic

The five hypotheses are evaluated with pre-specified comparisons between nominal, single-fault, and combined-fault conditions and, where independent outputs are available, between conventional, partially enabled, and complete fusion configurations. Accuracy, fault-management, integrity, risk, and authority outcomes are treated as distinct evidence domains. A hypothesis is regarded as supported only when the corresponding effect is directionally consistent, operationally material, and accompanied by an uncertainty interval or a complete event count; a favorable single trajectory is not sufficient evidence.

The hypotheses and the evidence required for their assessment are formalized in Table 3. Each row separates the scientific claim, the comparison that can actually be made with the recorded dataset, and the evidence needed for acceptance, thereby preventing a result from being supported by a metric that does not test the stated mechanism.

Table 3 links every hypothesis to a measurable outcome and prevents post hoc reinterpretation. It also distinguishes direct evidence, such as independently labelled FDI performance, from partial evidence that still requires estimator reruns or additional probabilistic calibration. The direct/partial distinction is especially important for H4 and H5: within-run risk separation and recorded authority states can be evaluated, but full probability calibration and a matched fixed-policy counterfactual cannot be inferred from the same records. Table 3 therefore functions as a prospective crosswalk linking the Methods, Results, and Discussion sections.

## 4. Proposed Quality- and Integrity-Aware Fusion Method

### 4.1. Preprocessing, Calibration, and Time Synchronization

Preprocessing preserves the separation between raw measurements, calibration parameters, quality indicators, and estimator outputs. Intrinsic sensor calibration is applied to cameras, LiDAR, radar, ultrasonic channels, and load cells; rigid-body extrinsic calibration maps each sensor frame into the vessel body frame; and the surveyed transformation between the navigation and berth frames provides the reference geometry for relative docking states. RTK-GNSS antenna lever arms, IMU/gyro alignment, camera lens distortion, radar/LiDAR boresight, ultrasonic beam orientation, and load-cell zero/span coefficients are applied before fusion. The consistency requirements of vision-aided inertial navigation and robust visual–inertial estimation motivate the explicit calibration, timestamp, and covariance checks applied before fusion [44,45].

The plausibility layer removes only observations that violate hard physical or protocol constraints, such as invalid GNSS fixes, non-finite values, impossible ranges, duplicate timestamps, stale messages, load-cell saturation, or inconsistent sequence numbers. Statistical outliers are not deleted at this stage because they are needed by the FDI module. Missing measurements remain explicitly missing; zero is retained only when it is a physically measured zero. The original flags, device timestamps, corrected timestamps, and calibration version are preserved for auditability.

Time synchronization uses the UTC reference and the channel-specific clock source. High-rate propagation is driven by inertial timestamps, while all exteroceptive and operational measurements are inserted according to their corrected times. The synchronization procedure is validated against the recorded reference uncertainty. Coordinate transformations are applied after temporal alignment so that cross-sensor residuals compare states referring to the same physical epoch. This ordering is important in confined-water maneuvers, where even a short delay can convert a valid range observation into an apparent spatial inconsistency.

### 4.2. Quality-Aware Covariance Adaptation

Fixed measurement covariances are inadequate when signal quality changes with satellite visibility, multipath, clutter, precipitation, glare, occlusion, packet age, or load-cell condition. The proposed method preserves the calibrated covariance structure of each modality but scales it according to the current trust coefficient.

(10) $$R_{i,k}^{eff}=\alpha_{i,k}R_{i,0},\;\alpha_{i,k}=clip\;\left(\gamma_{i,k}^{-2},\alpha_{i}^{\min},\alpha_{i}^{\max}\right),$$

where $R_{i,k}^{eff}$ is the effective covariance used in the measurement update; $R_{i,0}$ is the nominal calibrated covariance; $\alpha_{i,k}$ is the covariance scale; and $\alpha_{i}^{\min}$ and $\alpha_{i}^{\max}$ are modality-specific lower and upper limits. The inverse-square mapping makes the standard deviation approximately inversely proportional to trust. The lower bound prevents the filter from becoming more confident than the calibration supports, and the upper bound prevents numerical ill-conditioning before the channel is formally excluded.

To avoid abrupt changes caused by a single noisy indicator, the scale is smoothed and rate-limited.

(11) $${\bar{\alpha }}_{i,k}=\mathrm{clip}\;\left[(1-\lambda_{i}){\bar{\alpha }}_{i,k-1}+\lambda_{i}\alpha_{i,k},\;{\bar{\alpha }}_{i,k-1}-\Delta_{i}^{-},\;{\bar{\alpha }}_{i,k-1}+\Delta_{i}^{+}\right],$$

where ${\bar{\alpha }}_{i,k}$ is the applied covariance scale; $\lambda_{i}$ is the smoothing coefficient; and $\Delta_{i}^{-}$- and $\Delta_{i}^{+}$ are the maximum permitted downward and upward changes per accepted observation. Upward inflation is allowed to proceed faster than downward recovery so that the estimator reacts rapidly to degradation but restores trust conservatively. The applied scale is stored in the experimental data-quality table, enabling direct comparison between sensor condition, trust, covariance inflation, and navigation error.

Covariance adaptation is applied independently to each sensor block. Cross-sensor agreement influences trust but does not force identical weights across modalities. Consequently, a radar channel can remain useful for relative obstacle geometry when GNSS is degraded, while an environmentally impaired camera can be down-weighted without suppressing LiDAR or ultrasonic ranges. This modality-specific treatment is central to preserving observability under partial failures.

**Figure 4:**
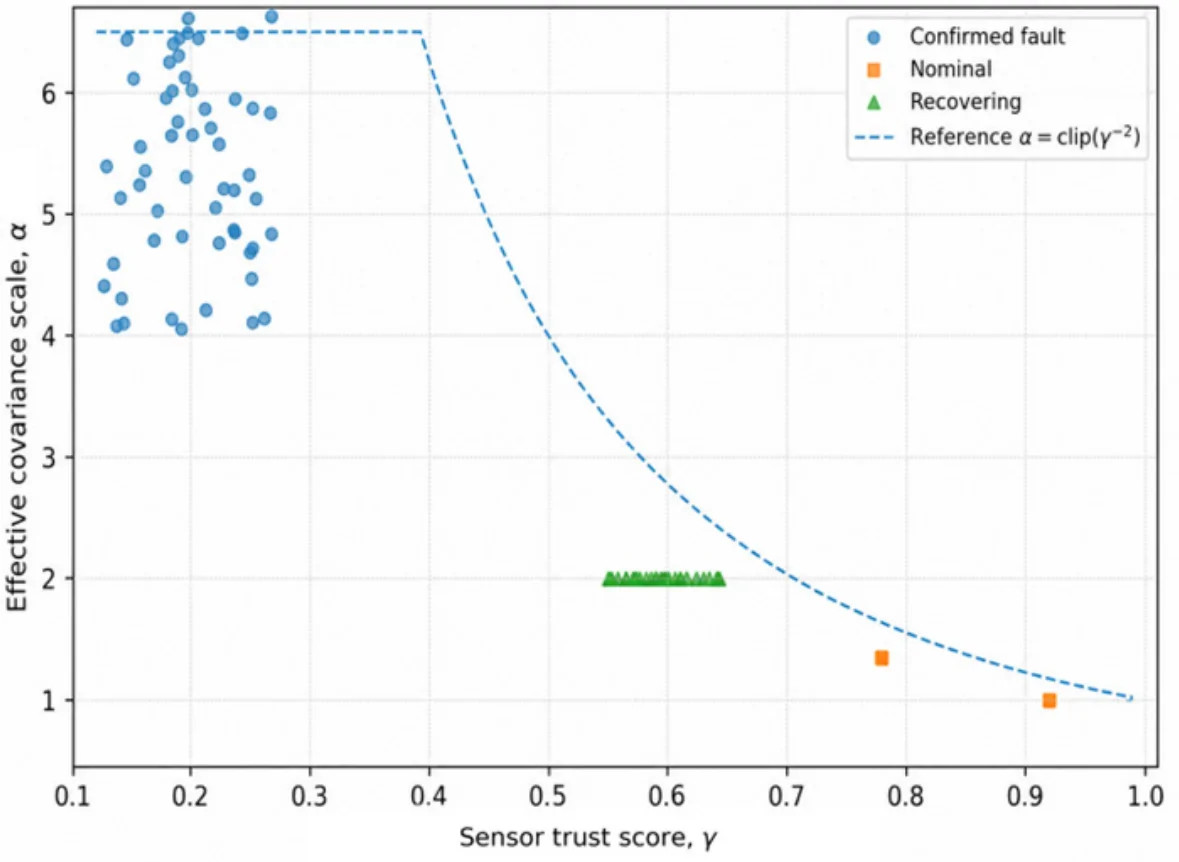
Observed relationship between the sensor trust coefficient *γ* and the effective covariance scale *α* for 480 quality records; both quantities are dimensionless.

The measured relationship between sensor trust and the effective covariance scale is shown in Figure 4. The scatter points are separated by FDI mode, and the dashed inverse-square reference indicates the nominal quality-to-information relationship before clipping and temporal smoothing.

The points in Figure 4 occupy three mode-dependent bands. Nominal records remain near the unit covariance scale, recovering records occupy an intermediate band, and confirmed faults approach the upper inflation range. The banded structure follows from the bounded sensor-mode controller and the rate-limited covariance law; it is not quantization of the physical measurements. The upper plateau is the imposed covariance-scale cap used to avoid numerical ill-conditioning before exclusion, while the intermediate recovery band shows that a channel does not regain nominal information weight immediately after a fault.

### 4.3. Fault Detection, Isolation, and Mode Classification

Fault detection combines model-based innovation tests with cross-sensor consistency and temporal persistence. For each candidate update, the innovation and its covariance are

(12) $$\nu_{i,k}=z_{i,k}-h_{i}(x_{k}^{-}),\;S_{i,k}=H_{i,k}P_{k}^{\text{-}}H_{i,k}^{T}+R_{i,k}^{\mathrm{eff}},$$

where $\nu_{i,k}$ is the innovation; $H_{i,k}$ is the measurement Jacobian evaluated at the predicted state; and $S_{i,k}$ is the innovation covariance. The use of $R_{i,k}^{\mathrm{eff}}$ ensures that an observation is tested against the uncertainty implied by its current quality. Cross-sensor residuals are evaluated in a common physical coordinate, such as horizontal position, relative berth distance, target range-bearing, heading, or line tension.

The principal scalar consistency statistic is the normalized innovation squared. Its threshold depends on the measurement dimension and the sensor-specific false-alarm allocation.

(13) $$D_{i,k}^{2}=\nu_{i,k}^{T}S_{i,k}^{-1}\nu_{i,k},c_{i,k}=max\left\{0,c_{i,k-1}+1\left[D_{i,k}^{2}>\chi_{d_{i},1-\alpha_{i}}^{2}\right]-1\left[D_{i,k}^{2}\leq \chi_{d_{i},1-\alpha_{i}}^{2}\right]\right\}$$

where $D_{i,k}^{2}$ is the normalized innovation squared (NIS), calculated from the innovation vector $\nu_{i,k}$ and its covariance matrix $S_{i,k}$; $d_{i}$ is the dimension of the observation vector; $\chi_{d_{i},\;1-\alpha_{i}}^{2}$ is the chi-square threshold corresponding to the significance level $\alpha_{i}$; and $c_{i,k}$ is a persistence counter, which is incremented when the innovation test fails and decremented after acceptable observations (bounded below by zero). The variables $n_{F,i},\;n_{R,i},\;m_{i,k}$ denote the confirmation threshold, recovery threshold, and current sensor mode (N: nominal, S: suspected, F: confirmed fault, R: recovering), respectively, which govern the mode transition logic based on the value of $c_{i,k}$. In the counter recursion, $c_{i,k-1}$ is the previous persistence-counter value, and 1[·] denotes the indicator function.

Fault isolation uses modality-specific signatures. Bias and drift are distinguished from short spikes by persistence and residual slope; dropout and packet loss are identified from availability and sequence continuity; delay and time offset are identified from timestamp residuals; GNSS multipath is associated with position innovation, correction age, fix state, and satellite geometry; radar clutter and AIS latency are separated by track consistency and message age; LiDAR/camera degradation is linked to feature confidence and environmental suitability; and load-cell faults are tested against neighboring line behavior, thrust demand, and physical tension bounds. The fuzzy fault-tolerant fusion concept reported by Zhu et al. [10] provides a relevant precedent, while the present implementation retains explicit quality, mode, and integrity states for every sensor.

The experimental fault table provides an independent label for the onset, duration, magnitude, and restoration of each controlled degradation. Detection probability and latency are therefore calculated from registered fault intervals rather than inferred retrospectively from navigation error. This separation supports reproducible assessment of false alarms, missed detections, and recovery performance.

**Figure 5:**
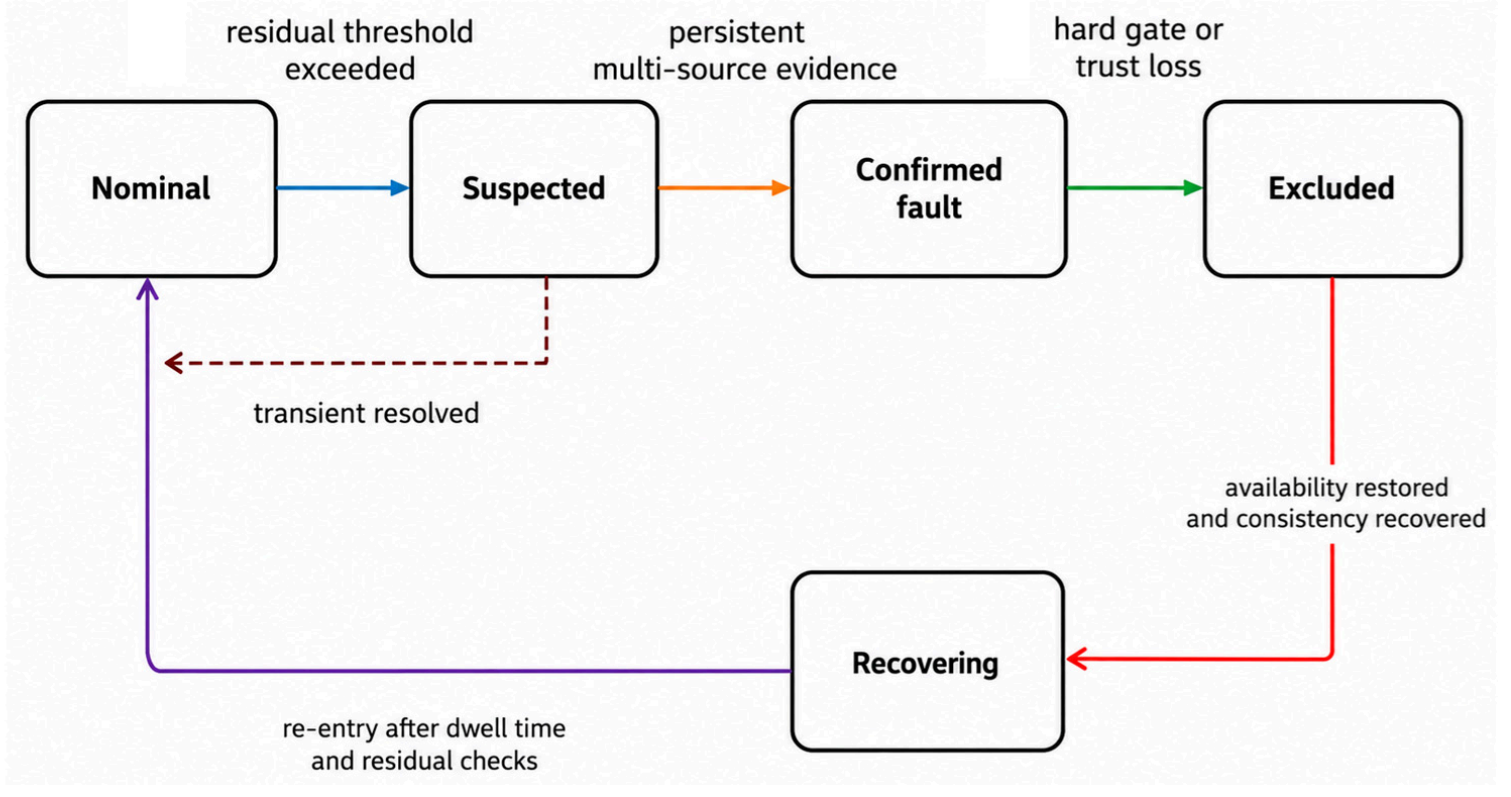
Fault-detection, isolation, exclusion, recovery, and controlled re-entry state logic.

The fault-management logic that converts residual evidence into exclusion and controlled recovery is summarized in Figure 5. The diagram separates evidence accumulation, hard-gate entry, temporary exclusion, and re-entry conditions so that transient outliers and persistent faults do not follow the same path.

Figure 5 makes the recovery safeguards explicit. A transient innovation excursion may return from the suspected state without exclusion, whereas confirmed faults require trust recovery, residual consistency, restored availability, and the prescribed dwell time before the channel can re-enter the active set. The dashed transient-resolution branch avoids unnecessary loss of redundancy, whereas the full exclusion–recovery loop protects the estimator from premature readmission. These asymmetric transition requirements explain why detection can be rapid while recovery is deliberately slower.

Formal mode-transition pseudocode (per sensor i): initialize $m_{i}=N$, $n_{i}^{bad}=0$, and $n_{i}^{good}=0$. For each corrected observation, first apply the hard validity and availability gates. If a hard fault is present, set $m_{i}=F$ and $n_{i}^{good}=0$. Otherwise, evaluate the NIS and cross-sensor consistency tests. If either soft test fails, increment $n_{i}^{bad}$ by one and reset $n_{i}^{good}=0$; if both pass, increment $n_{i}^{good}$ by one and decrement $n_{i}^{bad}$ toward zero. Transition N → S after the first persistent soft-failure indication; transition S → F when $n_{i}^{bad}\geq n_{conf}$; transition S → N when $n_{i}^{good}\geq n_{clear}$. From F, enter R only after availability and hard-gate validity are restored; transition R → N when $n_{i}^{good}\geq n_{rec}$; transition R → F if a hard fault reappears or $n_{i}^{bad}$ again reaches $n_{conf}$. The counters $n_{conf}$, $n_{clear}$, and $n_{rec}$ are configuration parameters and are applied independently to each sensor channel.

Calibration values/initial settings: the initial NIS significance level is $\alpha_{NIS}=0.01$ (99% chi-square consistency gate); soft-fault confirmation uses $n_{conf}=3$ consecutive failed consistency checks; suspected-to-nominal clearance uses $n_{clear}=3$ consecutive acceptable checks; and controlled recovery uses $n_{rec}=5$ consecutive acceptable checks before R → N readmission. Hard protocol/availability faults retain immediate exclusion.

### 4.4. Fault-Tolerant Multisensor Fusion and Reconfiguration

The fusion core is an error-state extended Kalman filter with sequential asynchronous updates. This structure was selected because the navigation dynamics and sensor transformations are nonlinear, whereas each arriving measurement block is comparatively small and is associated with an explicit covariance. For an admitted sensor, Equation (14) defines the Kalman gain and the manifold-consistent state correction. The sequential architecture is compatible with tightly coupled LiDAR–inertial smoothing and direct LiDAR–inertial odometry, which demonstrate the value of retaining modality-specific residuals and update timing [46,47].

(14) $$K_{i,k}=P_{k}^{-}H_{i,k}^{T}S_{i,k}^{-1},\;x_{k}^{+}=x_{k}^{-}⊞K_{i,k}\nu_{i,k},$$

where $K_{i,k}$ is the Kalman gain and boxplus denotes injection of the estimated error state into the nonlinear nominal state, including a proper attitude update. Each sensor block is processed using its effective covariance, so quality adaptation changes the information contribution without altering the physical measurement model.

The active measurement set is reconstructed at every event.

(15) $$A_{k}=\left\{i:a_{i,k}=1,\;m_{i,k}\notin \{F\},\;\gamma_{i,k}\geq \gamma_{i}^{\min},\;{\hat{\delta }}_{i,k}\leq \delta_{i}^{\max}\right\},$$

where $A_{k}$ is the active sensor set; $a_{i,k}$ is the availability state; $\gamma_{i}^{\min}$ is the minimum admission trust; $\delta_{i}^{max}$ is the maximum admissible absolute time offset; and the confirmed-fault state *F* prevents assimilation. Suspected sensors remain available with increased covariance unless a hard safety rule requires immediate exclusion. This soft-to-hard progression avoids unnecessary loss of redundancy while still preventing a persistent faulty channel from dominating the estimate. In Equation (15), $m_{i,k}$ is the current FDI mode and $\delta_{i,k}$ is the absolute corrected time offset for sensor i.

The posterior covariance is updated in Joseph form to preserve symmetry and positive semidefiniteness under large covariance scales and repeated reconfiguration.

(16) $$P_{k}^{+}=(I-K_{i,k}H_{i,k})P_{k}^{-}{\left(I-K_{i,k}H_{i,k}\right)}^{T}+K_{i,k}R_{i,k}^{\mathrm{eff}}K_{i,k}^{T},$$

where *I* is the identity matrix. The update is applied sequentially for all *i* in *A_k_* at their corrected timestamps. When a critical absolute-position channel is unavailable, the estimator continues with inertial, heading, radar/LiDAR berth or shoreline constraints, visual observations, actuation feedback, and environmental states. When exteroceptive channels are unavailable during final docking, the system retains only the sensor combinations that satisfy the phase-specific observability and integrity requirements; otherwise, the solution is marked unavailable and the decision layer requests a protective hold or manual takeover. A broader camera–LiDAR–IMU survey likewise identifies calibration, synchronization, uncertainty representation, and degraded-modality handling as central determinants of fusion robustness [48].

Re-entry is controlled rather than instantaneous. A recovering sensor first passes plausibility and timestamp checks, then accumulates $n_{R,i}$ consistent innovations, and finally returns with an initially inflated covariance that decays according to Equation (11). The resulting mode sequence—nominal, suspected, confirmed fault, recovering, and nominal—is recorded in the data-quality table and forms the basis for the reported detection and recovery latencies. The controlled recovery logic is also consistent with stochastic reliability analysis and diagnostic-parameter approaches used for autonomous technical systems [49].

**Figure 6:**
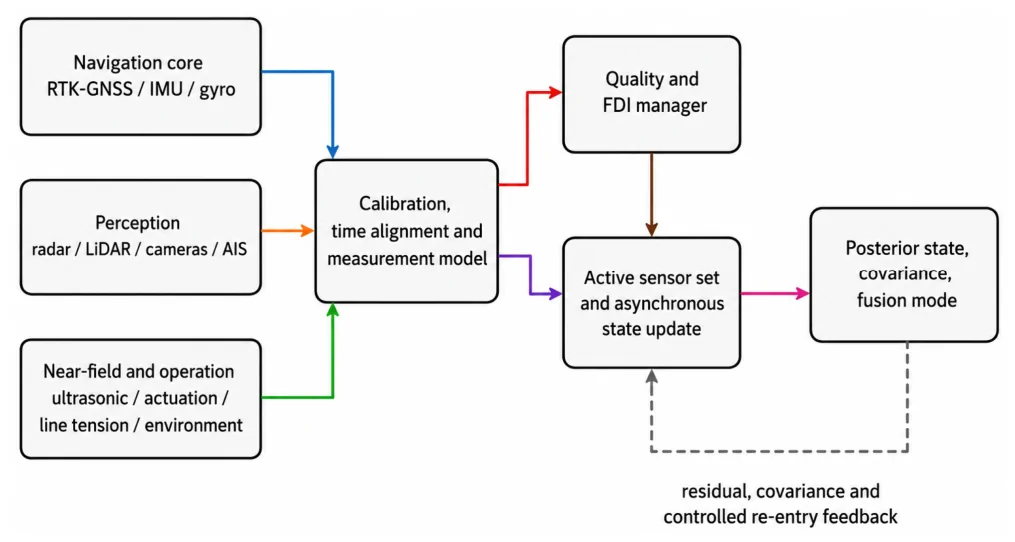
Heterogeneous sensor-fusion and active-set reconfiguration architecture.

The interaction among local sensing groups, calibration/time alignment, the FDI manager, and the active-set estimator is shown in Figure 6. The figure complements Figure 1 by resolving the fusion layer into its modality-specific inputs and its residual/covariance feedback path.

As shown in Figure 6, individual modalities are not fused indiscriminately. The quality and FDI manager determine the active set, while posterior residuals and covariance provide feedback for subsequent weighting and controlled sensor re-entry. The architecture therefore preserves two levels of modularity: sensor-specific preprocessing remains local, while admission and posterior consistency are coordinated globally. This design permits one modality to be isolated without disabling unrelated observables and makes the reason for each active-set change auditable.

### 4.5. Integrity Monitoring and Protection Levels

Integrity monitoring determines whether the estimator can safely support the current operational phase. It is evaluated separately for horizontal position, heading, berth-relative distance, berth-relative angle, and line tension. A hazardously misleading information (HMI) event occurs when the actual error exceeds the phase-specific alert limit while the protection level still indicates that the solution is available.

(17) $$H_{c,k}=\left\{|e_{c,k}|>AL_{c,s(k)}\right\}\cap \left\{PL_{c,k}\leq AL_{c,s(k)}\right\},$$

where $H_{c,k}$ is the hazardously misleading information (HMI) event for monitored component *c*; $e_{c,k}$ is the estimation error, available from the independent reference during evaluation; $L_{c,s(k)}$ is the alert limit for component c in phase s(k); and $PL_{c,k}$ is the calculated protection level. During operation, the actual error is unknown; therefore, safety depends on the conservatism and calibration of *PL* rather than on accuracy alone.

The protection level combines the covariance-projected standard deviation, a bounded residual-bias term, and an explicit contribution from unresolved fault probability.

(18) $$PL_{c,k}=K_{c,s}\sqrt{a_{c}^{T}P_{k}^{+}a_{c}}+b_{c,k}^{\max}+\sum_{i\in A_{k}}p_{i,k}^{F}\Delta_{c,i,k},$$

In Equation (18), $K_{c}$ is the phase- and component-specific integrity multiplier applied to the covariance-projected standard deviation. The term $p_{i,k}^{F}$ is not treated as a Bayesian posterior probability in the present implementation; it is a conservative unresolved-fault hypothesis weight associated with the current FDI mode and persistence state. $\Delta_{c,i,k}$ is the worst-case effect of the corresponding sensor-fault hypothesis on monitored component c. It is obtained from the registered fault magnitude or calibration envelope propagated through the corresponding measurement sensitivity and therefore has the same physical units as component c (metres for horizontal/relative position, degrees for heading/relative angle, and kilonewtons for line tension). The product $p_{i,k}^{F}\Delta_{c,i,k}$ is consequently an additive conservative fault-exposure allowance rather than a second covariance term. Here, $g_{c}$ is the component-selection/sensitivity vector used to project the posterior covariance onto monitored component c, and $b_{c,k}^{res}$ is the bounded residual-bias allowance.

The experimental configuration uses horizontal-position and heading alert limits of 2.0 m and 5.0 degrees during restricted-channel transit, and berth-relative position and heading limits of 0.5 m and 3.0 degrees during final docking. Approach limits vary continuously between transit and docking values according to remaining berth distance and closing speed. Line-tension availability is assessed against the verified operating limit of each instrumented line. A solution is declared available only when all safety-critical protection levels for the current phase remain below their alert limits.

Calibration values/initial settings: $K_{c}=5.62$ is used as the conservative initial protection-level expansion factor for each safety-critical monitored component; this value follows the maritime integrity assessment reported by Zalewski [13], where k=5.62 corresponds to an integrity-risk order of $1.39\times 10^{-7}$ per statistically independent epoch. The unresolved-fault weights are initialized heuristically as $p_{i,k}^{F}=0.01$ in nominal mode, $p_{i,k}^{F}=0.10$ in suspected mode, and $p_{i,k}^{F}=0.05$ in recovering mode; a confirmed-fault sensor is excluded from the active set rather than assigned an active $p_{i,k}^{F}$. $\Delta_{c,i,k}$ is defined as the worst-case component-space fault envelope. When a sensor-specific registered/calibrated envelope is unavailable during transfer or replay setup, the conservative first-pass defaults are 0.50 m for horizontal position, 1.0 degrees for heading, 0.10 m for berth-relative distance, 1.0 degrees for berth-relative angle, and 5.0 kN for line tension.

Integrity is evaluated empirically by comparing protection levels with the independent reference errors at the 480 synchronized epochs. Coverage, uncovered-error count, phase-specific continuity, and hazardously misleading information events are reported. This evaluation distinguishes a conservative system, which may temporarily lose availability, from an unsafe system that remains available while its error exceeds the alert limit. For terminology, a protection-level non-envelopment occurs whenever $|e_{c,k}|>{PL}_{c,k}$. This is reported as an integrity-bound failure even if the error remains below the phase alert limit. An HMI-event is reserved for the operationally hazardous case in which the solution remains declared available while the relevant alert-limit requirement is violated.

**Figure 7:**
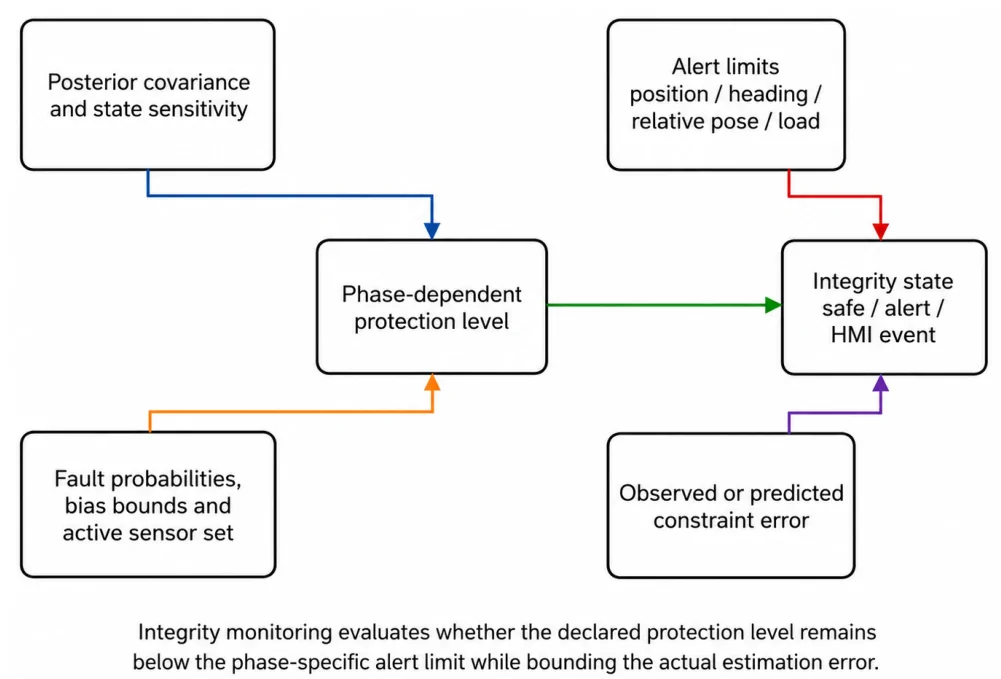
Propagation of covariance and fault information into protection levels and integrity states.

The mechanism by which posterior uncertainty and diagnosed fault information are combined to form the integrity decision is presented in Figure 7. The diagram distinguishes covariance-derived uncertainty, bounded bias/fault exposure, phase-specific alert limits, and the observed or predicted constraint error.

Figure 7 clarifies the distinction between accuracy and integrity. A valid integrity state requires the protection level to remain below the phase-specific alert limit while still bounding the actual or predicted error; a numerically precise point estimate alone is therefore insufficient. The converging paths show that an integrity state is not determined by covariance alone: unresolved fault effects and operational alert limits are co-determinants. This is important during docking, where a modest absolute error can be unacceptable under a tight local limit, while a larger transit error may remain operationally tolerable.

### 4.6. Navigation and Mooring Risk Propagation

The decision layer receives a distribution of the current state rather than a single deterministic estimate. Phase-specific hazard functions define violation of the channel corridor, unsafe closest-point-of-approach conditions, excessive berth closing speed or contact energy, and mooring-line overload. State covariance, unresolved fault weights, and short-horizon disturbance uncertainty are propagated through these functions.

(19) $$R_{k}^{\left(s\right)}=\frac{\sum_{j=1}^{J_{s}}w_{j,s}C_{j,s}\;\mathrm{Pr}\;\left[g_{j,s}(X_{k:k+H})>0|Z_{1:k}\right]}{\sum_{j=1}^{J_{s}}w_{j,s}C_{j,s}},$$

where $R_{s}$ is the normalized composite risk in phase s; $J_{s}$ is the number of active hazard functions; $w_{j,s}$ is the phase-dependent relevance weight; $C_{j,s}$ is the normalized consequence factor; $g_{j,s}$(·) is positive when hazard constraint j is violated; $X_{k:k+H}$ is the predicted state sequence over horizon H; and $Z_{1:k}$ denotes all accepted measurements up to the current epoch. The conditional violation probability is obtained by sigma-point or Monte Carlo propagation for nonlinear berth-contact and CPA constraints, and by a Gaussian closed form for linearized corridor, distance, heading, and line-load constraints.

During transit, channel-boundary and collision terms dominate. During approach, the weights shift toward berth-relative distance, lateral velocity, yaw rate, and stopping margin. During final docking, the principal terms are contact probability, contact energy, minimum clearance, and relative heading. During line securing, line overload, asymmetric loading, thrust-line interaction, and disturbance growth dominate. The composite risk stored in the experimental decision table is accompanied by separate channel-boundary, collision, berth-contact, line-overload, and integrity-risk fields, which permits the cause of an alert to be reconstructed.

Consequence weights do not substitute for regulatory or class-society safety criteria. They provide a dimensionless ranking within the investigated operation and are calibrated so that an increase in the probability of any severe constraint violation cannot reduce composite risk. Sensitivity analysis is required when the method is transferred to a different vessel, berth geometry, line arrangement, or operational envelope. No global sensitivity analysis of $w_{j,s}$ and $C_{j,s}$ was performed in the completed campaign. Because the composite score is a weighted sum of hazard probabilities, authority-mode boundaries are most parameter-sensitive for epochs lying close to the rule thresholds; states well inside a mode region are less sensitive to moderate weight perturbations. Accordingly, the reported authority-state frequencies are conditional on the calibrated policy and are not claimed to be invariant to alternative weight sets.

Calibration values/initial settings: when a phase-specific risk-priority matrix has not yet been re-estimated, the hazard weights are initialized uniformly as $w_{j,s}=1/J_{s}$ and then renormalized to sum to one. Initial normalized consequence factors are $C_{j,s}=1.0$ for severe safety constraints, $C_{j,s}=0.60$ for moderate constraints, and $C_{j,s}=0.30$ for advisory constraints. For transfer studies, a one-at-a-time ±20% perturbation of each weight followed by renormalization is the recommended initial sensitivity envelope.

### 4.7. Risk-Adaptive Human–Machine Authority Allocation

Authority is selected from five ordered modes: information only, recommendation, supervised automatic action, protective intervention, and manual takeover. The choice depends jointly on predicted operational risk, integrity risk, operator burden, and the cost of switching from the current mode.

(20) $$\begin{array}{l} A_{k}^{*}=\arg\min_{A\in M}\left\{{\hat{R}}_{k}(A)+\lambda_{I}I_{k}+\lambda_{H}L_{H}(A)+\lambda_{S}C_{\mathrm{sw}}(A,A_{k-1})\right\},\\ I_{k}=c^{\max}\mathrm{Pr}(H_{c,k}), \end{array}$$

where $A_{k}^{*}$ is the selected authority mode; *M* is the admissible mode set for the current phase; ${\hat{R}}_{k}(A)$ is the predicted residual operational risk if mode *A* is applied; $I_{k}$ is the maximum HMI probability across monitored components; $L_{H}(A)$ represents expected operator workload or required attention; $C_{\mathrm{sw}}(A,A_{k-1})$ penalizes unnecessary switching; and $\lambda_{I}$, $\lambda_{H}$, and $\lambda_{S}$ are non-negative policy weights. The optimization is implemented as a deterministic rule table with calibrated thresholds, because the five modes are discrete and must remain auditable. The variable $A_{k-1}$ denotes the authority mode applied at the preceding decision epoch.

Calibration values/initial settings: in the absence of a replay-identified authority policy, the initial non-negative policy weights in Equation (20) are set to $\lambda_{R}=0.50$ for residual operational risk, $\lambda_{I}=0.30$ for integrity risk, $\lambda_{W}=0.10$ for operator-burden/workload penalty, $\lambda_{S}=0.10$ for switching penalty. A conservative starting rule table uses a minimum dwell time of two synchronized decision epochs, upward-persistence of one epoch for transitions toward more conservative authority, and downward-persistence of two epochs before relaxation.

When risk and integrity risk are low, supervised automatic action is permitted. Moderate risk with adequate integrity generates a recommendation or supervised corrective action. High operational risk with strong integrity permits a bounded protective intervention, such as reducing approach speed or arresting lateral motion. Degraded integrity restricts autonomous authority even when geometric risk appears low. When high operational risk and degraded integrity coincide, the system requests manual takeover and enters a protective hold or minimum-risk maneuver until a valid sensor set is restored. The information-only mode is reserved for non-critical advisory states. In the completed dataset, recommendation, supervised automatic action, and protective intervention were the principal event-driven states; four nominal runs used a planned manual-reference policy, and two operator-event records documented actual takeover actions.

Two safeguards prevent unstable mode oscillation. First, upward transitions toward more conservative authority use lower persistence requirements than downward transitions. Second, every mode has a minimum dwell time unless an emergency threshold is crossed. Each recommendation contains an explanation code identifying the dominant hazard, the sensor or integrity condition that triggered the transition, the predicted time or distance margin, and the proposed action. These fields support operator verification and post-experiment traceability.

**Figure 8:**
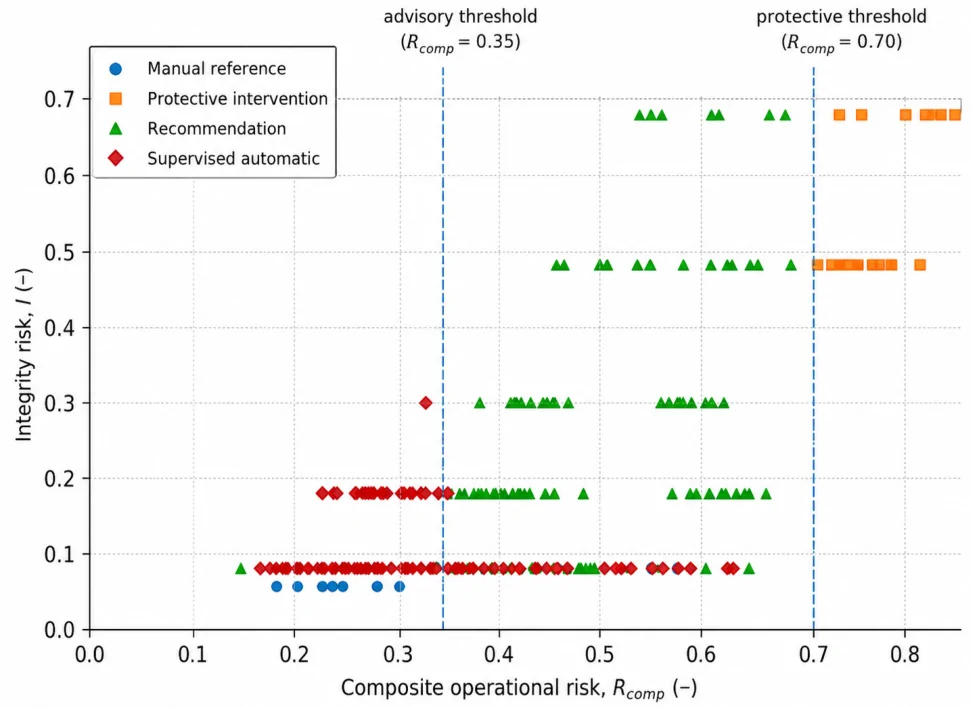
Observed joint distribution of composite operational risk and integrity risk by recorded authority state; “manual reference” denotes planned nominal runs and not an event-triggered takeover.

The experimental operating states in the joint composite-risk and integrity-risk plane are plotted in Figure 8. The scatter view is used because authority selection depends on both coordinates; a one-dimensional risk plot would conceal cases in which similar geometric risk is supported by different levels of measurement credibility.

Figure 8 shows that recommendation and supervised-automatic states occupy the intermediate-risk region, whereas protective intervention is concentrated beyond the higher composite-risk threshold. The planned manual-reference states overlap the nominal low-risk region and are therefore not evidence of risk-triggered takeover. Actual takeover actions are evaluated separately from the operator-event log. This distinction prevents the experimental baseline policy from being conflated with a safety response. The dashed advisory and protective thresholds explain the horizontal separation of authority states, while the vertical spread captures the integrity contribution. The remaining overlap is expected because hysteresis, dwell time, operational phase, and the previous authority state also enter the rule table.

### 4.8. Algorithmic Workflow and Computational Complexity

The complete method is event driven. Inertial propagation is executed at the native high rate, while each exteroceptive, cooperative, environmental, actuation, proximity, or line-load observation triggers its own quality assessment and conditional update. Integrity and risk are recomputed after every safety-relevant update and at the synchronized decision epochs used for experimental evaluation. The ordered processing sequence is formalized in Algorithm 1. A defining feature of the algorithm is the separation of hard rejection from soft degradation handling: only protocol-corrupt or physically invalid samples are discarded immediately, whereas plausible but low-confidence observations remain available to the diagnostic path and can be down-weighted, isolated, or admitted under an inflated covariance. This distinction preserves observability and diagnostic traceability during intermittent sensor degradation.
**Algorithm 1.** Event-driven quality- and integrity-aware fusion cycleInput: previous posterior state and covariance; buffered inputs; incoming measurement *z_i,l_*; calibration, quality, FDI, integrity, and authority parameters. 1. Correct the timestamp and reject only hard protocol or physical-invalidity cases. 2. Propagate the nominal state and error covariance to the corrected observation time. 3. Compute raw quality indicators, innovation, cross-sensor residuals, and the trust coefficient. 4. Update the covariance scale and sensor mode; classify the event as nominal, suspected, confirmed fault, or recovering. 5. If the sensor is admitted, perform the sequential error-state update in Joseph form; otherwise retain it for diagnostics. 6. Re-propagate to the current estimator time when an out-of-sequence update has been accepted. 7. Compute component-wise protection levels, solution availability, integrity risk, and phase-specific hazard probabilities. 8. Select the authority mode with hysteresis and minimum dwell time; issue the action and explanation code. 9. Store state, covariance, trust, FDI mode, protection levels, risk components, authority mode, and outcome fields. Output: integrity-bounded fused state and auditable human–machine decision-support state.

Algorithm 1 integrates estimation, diagnostics, integrity monitoring, risk assessment, and authority allocation within one auditable event cycle. Its principal importance is that the navigation estimate and the safety state are updated from the same corrected measurement event and posterior covariance. The admission branch prevents a confirmed faulty channel from contaminating the fused state, while the diagnostic-only branch retains the rejected observation for fault-persistence tests and controlled re-entry. The Joseph-form update preserves covariance symmetry and positive semidefiniteness, and the explicit re-propagation step maintains temporal consistency when an out-of-sequence observation is accepted. Consequently, each protection-level change, risk update, and authority transition can be traced to the corresponding sensor-quality evidence rather than inferred retrospectively. The algorithm is therefore important not only as an implementation recipe but as the reproducibility contract for the paper: every reported state, fault classification, integrity value, and authority decision can be reconstructed from the ordered event log and parameter set.

**Figure 9:**
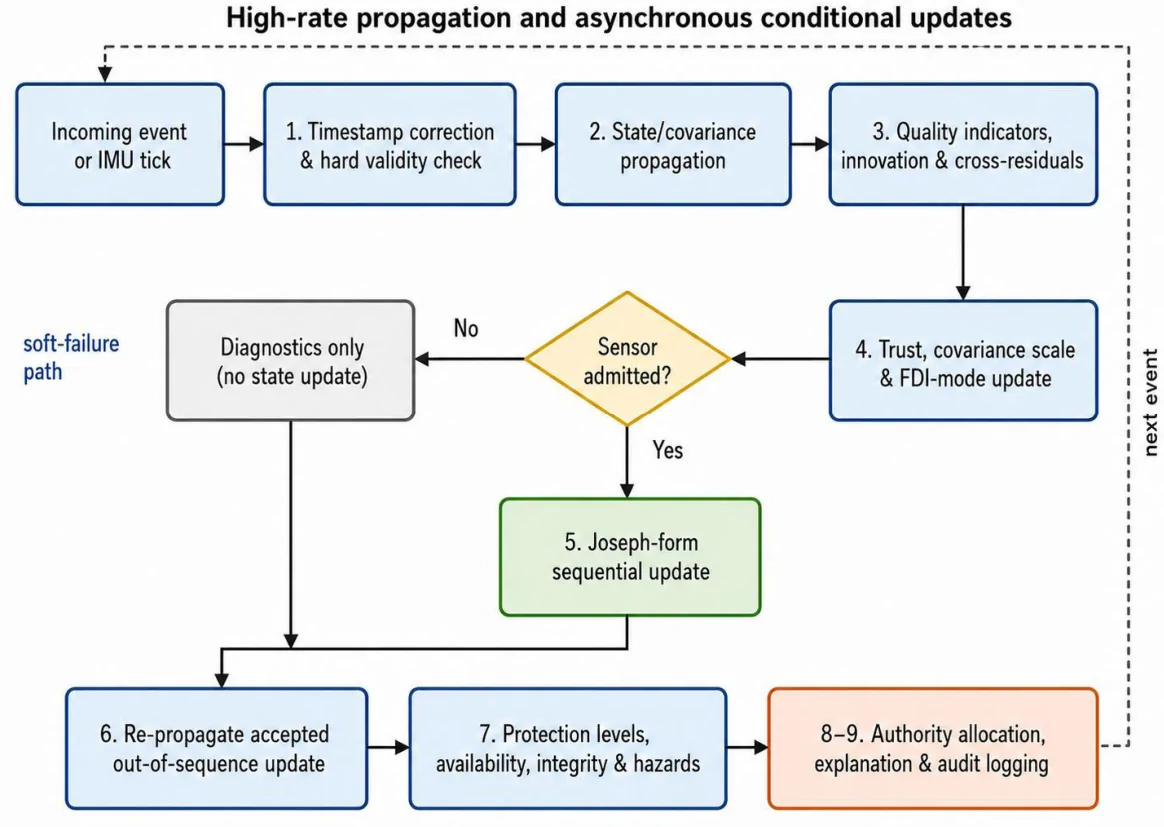
Event-driven implementation of Algorithm 1: timestamp correction and propagation; quality, innovation, and cross-residual evaluation; fault-aware sensor admission; Joseph-form update or diagnostic-only routing; re-propagation; integrity and hazard recalculation; authority allocation; and event-by-event audit logging.

The data, diagnostic, and decision paths defined in Algorithm 1 are summarized schematically in Figure 9. The diagram follows one measurement event from timestamp correction through the admission decision and shows both the state-update branch and the diagnostic-only soft-failure branch.

Figure 9 emphasizes three structural properties of the proposed method. First, asynchronous sensing is handled without forcing all channels into a common acquisition rate; every observation is processed at its corrected time. Second, degraded measurements follow a soft-failure pathway, so exclusion from the state update does not remove them from the diagnostic record. Third, integrity and authority are not periodic post-processing functions: they are recalculated after each safety-relevant update. The upper path represents accepted observations, whereas the lateral diagnostic branch preserves rejected evidence for persistence testing and controlled re-entry. This closed event loop is important in confined waters because a short-lived loss of sensor credibility can require an immediate change in protection level or control authority before the nominal trajectory leaves its admissible corridor.

For a state dimension $n_{x}$ and a measurement block of dimension $m_{i}$, one sequential update requires the matrix operations stated above; the dense covariance propagation is cubic in the state dimension, although the fixed sparsity of the navigation error model permits a lower practical cost. Because radar, LiDAR, camera, AIS, proximity, and line-load observations are processed as small blocks rather than one monolithic vector, the matrix inversion dimension remains $m_{i}$ rather than the sum of all sensor dimensions. Quality, FDI, protection-level, and rule-table calculations are linear in the number of active channels.

The architecture is compatible with native 100 Hz inertial propagation, 20 Hz GNSS/camera/proximity updates, 10 Hz radar/LiDAR updates, and 1 Hz AIS/environment updates without requiring acquisition-level synchronization. The recorded sensor-channel latency distribution characterizes communication and acquisition timing only. End-to-end estimator execution time, memory use, and worst-case decision latency were not logged independently; therefore, no processor-level real-time performance claim is made. Dedicated hardware profiling remains necessary before deployment or certification.

Reproducibility requires retaining the sensor calibration version, all covariance and threshold parameters, phase-transition logic, fault-state counters, integrity multipliers, risk weights, and authority-policy settings together with the processing-code version. These parameters are stored separately from the observations so that the 40 runs can be reprocessed under baseline and ablation configurations without altering the original measurement records. Diagnostic-parameter-based reliability studies of autonomous transport systems provide an additional precedent for retaining configuration and degradation-state metadata during reprocessing [50].

## 5. Experimental Platform, Dataset, and Validation Design

### 5.1. Experimental Vessel and Instrumentation

The experimental campaign used an instrumented research workboat designated RV-SEN02 for confidentiality. The recorded materials include sensor topology, manufacturer and model information, sampling rates, coordinate conventions, mounting coordinates, quality indicators, synchronized state estimates, independent reference measurements, controlled fault intervals, and decision-layer outputs. The platform uses UTC time, a local ENU navigation frame, a body-fixed frame, and multi-rate acquisition. Equipment serial numbers and the exact vessel and site identifiers are withheld. Quantitative claims are restricted to variables supported by documented measurements or calibration records. Raw observations, estimator outputs, reference states, and fault labels were stored separately to preserve an independent evaluation chain [51].

### 5.2. Test Area and Operational Scenarios

**Figure 10:**
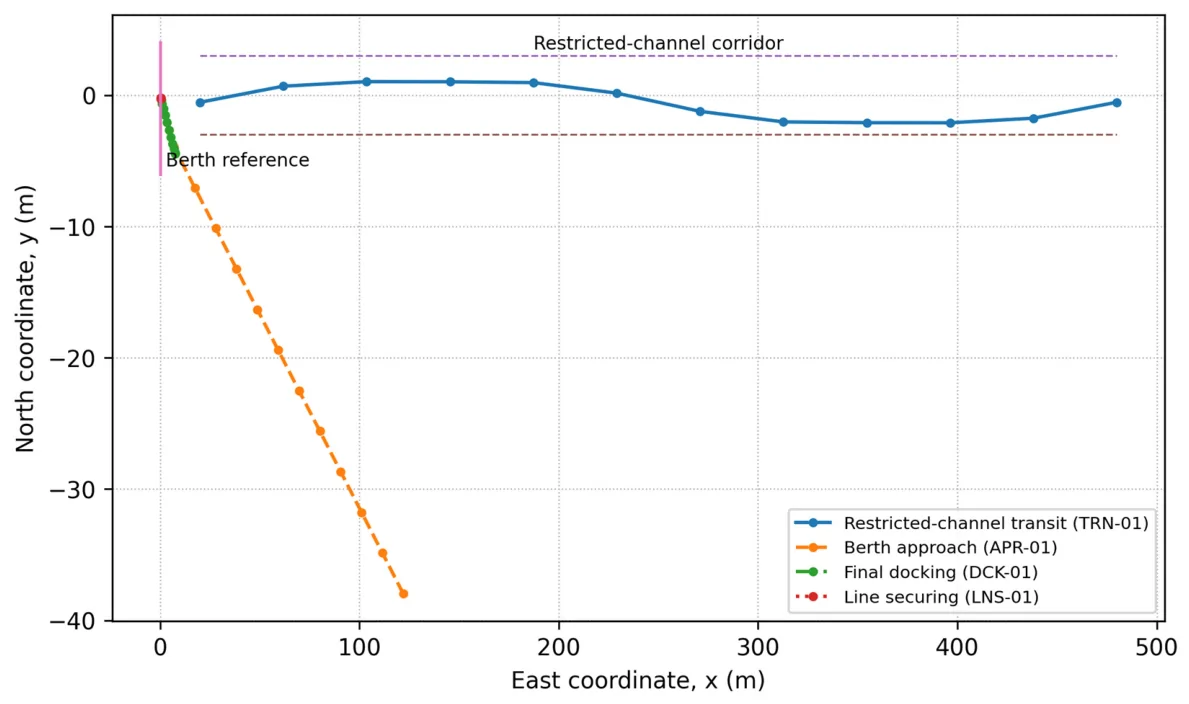
Representative nominal reference trajectories in the local ENU frame for restricted-channel transit, berth approach, final docking, and line securing; the dashed boundaries indicate the experimental channel corridor and the berth reference.

The campaign was conducted from 15 to 19 June 2026 as a five-day controlled confined-water experiment. The allocation of runs was fixed before acquisition: each operational phase contained six nominal runs, three single-degradation runs, and one combined-degradation run, giving 40 runs in total. This balanced design ensured equal phase coverage and was not intended to estimate natural fault frequency. Weather classes included calm, moderate, and adverse conditions, and traffic classes ranged from no traffic to dense traffic. Measurements are reported in a surveyed local ENU frame. For confidentiality and operational-security reasons, the exact berth identifier, public georeferenced site plan, and certified bathymetric chart are not reproduced. Figure 10 therefore presents the experimental trajectories in local coordinates and is not a navigational chart.

Figure 10 demonstrates the strong contraction of the operating envelope near the berth. The transit trajectory extends over hundreds of meters and is governed primarily by global position, heading, corridor clearance, and traffic state. The approach trajectory converges toward the surveyed berth reference, while docking and line-securing motion is confined to a meter-scale region. This scale transition explains why ultrasonic, LiDAR, camera, actuation, and line-tension channels gain relative importance in the final phases. The figure is therefore not only a site illustration; it provides the geometric basis for phase-dependent alert limits and sensor weighting.

The allocation of completed runs among operational phases and degradation classes is reported in Table 4. The matrix allows the reader to verify the balanced phase design, the 24/12/4 split among nominal, single-fault, and combined-fault runs, and the specific degradation families represented in each phase.

The balanced structure in Table 4 provides equal phase coverage and a consistent nominal/single-fault/combined-fault pattern. Nevertheless, individual fault mechanisms have limited repetition, so fault-specific estimates are interpreted within the completed campaign rather than as universal failure probabilities. Equal phase counts prevent one maneuvering stage from dominating the aggregate dataset, while the repeated 6/3/1 structure supports direct completeness checks across phases. The final column also links each run class to the state component most likely to be affected, which is necessary for interpreting channel-specific rather than universal error growth.

The completed experimental campaign provides balanced phase coverage with intentionally concentrated fault exposure. The number of repetitions differs among individual fault mechanisms and severity levels; therefore, numerical comparisons are reported using run-level summaries, confidence intervals, and fault-family stratification rather than interpreted as population equipment-failure probabilities.

### 5.3. Ground Truth, Calibration, and Synchronization Validation

Each of the 40 runs contains 12 synchronized reference epochs, yielding 480 ground-truth rows. The navigation reference is an independent RTK solution with a calibrated horizontal-position accuracy of 0.008 m and heading accuracy of 0.04°. Approach and docking records additionally contain berth distance and relative angle, whereas line-securing records contain reference line tension. Synchronization uncertainty is recorded for every reference epoch, and all reference rows passed the validity criteria. These reference channels provide the metrological basis for the position, heading, relative-pose, and line-load error calculations reported below. Agreement with the reference is assessed as a measurement-comparison problem rather than by correlation alone, and run-level uncertainty is quantified by resampling methods [52,53].

### 5.4. Controlled Fault-Injection Protocol

Sixteen controlled fault events were registered, four in each operational phase. Transit faults comprise GNSS multipath with a 3.2 m magnitude, GNSS dropout, IMU drift of 0.8°/min, and a combined GNSS bias–delay case. Approach faults include LiDAR occlusion, camera occlusion, radar clutter, and a combined LiDAR degradation with berth-range bias. Docking faults include ultrasonic dropout, camera occlusion, radar delay of 180 ms, and a combined ultrasonic–camera loss. Line-securing faults include an 8 kN tension bias, a 250 ms thrust delay, a 25 kN tension spike, and a combined line-tension/visual degradation. Each event has an independent fault identifier, onset and end time, severity level, waveform class, expected detector, and restoration flag. The explicit preservation of waveform, magnitude, onset, duration, and sensor identity follows signal-oriented diagnostic modelling practice [54].

### 5.5. Operational Configurations and Fault-Study Variants

Table 5 defines how the experiment identifiers map to authority policies and fault-study roles. This clarification is necessary because the codes B1–B5, P, and A1–A3 could otherwise be mistaken for independent estimator algorithms, although several configurations use the same fused-solution source.

Table 5 clarifies that the encoded method and policy identifiers represent operational configurations available in the dataset. They are not treated as independent algorithmic ablations unless a separate estimator output exists for the corresponding configuration. Consequently, comparisons among these codes support operational-policy and fault-family interpretation, but not component-removal causality. The table establishes the boundary between evidence contained in the current dataset and the replay experiments required for a formal ablation study.

### 5.6. Performance Metrics and Statistical Analysis

The quantitative criteria used throughout Section 6 are consolidated in Table 6. Metrics are grouped by evidence domain so that estimation accuracy, local docking performance, diagnostic classification, integrity, risk, authority behavior, and input-channel timing are not collapsed into a single performance score.

Table 6 ensures that accuracy, integrity, risk, operator response, and sensor-input timing are evaluated separately. This separation is necessary because a small mean error does not by itself establish fault-detection quality, alert calibration, or safe authority allocation. The reporting column also specifies when uncertainty intervals, event counts, or calibration targets are required. In particular, acquisition/communication latency is kept separate from estimator execution time, and Brier/ECE metrics are explicitly conditional on the availability of binary outcomes.

Primary errors are calculated at synchronized evaluation epochs and summarized at run level to reduce pseudo-replication. Horizontal error is the Euclidean distance between fused and reference ENU coordinates; heading error is the absolute wrapped angular difference. RMSE, MAE, maximum absolute error, and protection-level coverage are reported. Berth-distance, relative-angle, and line-tension errors are evaluated against the corresponding reference fields. Fault-detection performance uses independently registered fault windows and reports sensitivity, specificity, event-log detection latency, and controlled-recovery latency. Authority evaluation reports alert adjudication, unsafe-autonomy events, unnecessary takeovers, mode switches, aborted decision epochs, and operator response latency. Run-level bootstrap confidence intervals use resampling with replacement. Because all injected-fault runs were acquired in the adverse weather class, whereas nominal runs were acquired in calm or moderate classes, and because authority policies also differed across nominal runs, nominal/single/combined condition comparisons are treated as descriptive. The primary inferential comparison for risk response uses n = 16 paired run-level observations, each formed from fault-active and fault-inactive intervals within the same fault run, and a two-sided Wilcoxon signed-rank test at α = 0.05. This separation of accuracy, reliability, and diagnostic evidence is consistent with broader reviews of technical-system and transport reliability methods [55].

## 6. Results

### 6.1. Dataset Composition and Sensor Data Quality

The experimental dataset contains 40 completed runs with no excluded sequence. Each phase contributes ten runs and 120 synchronized navigation, perception, actuation, reference, risk, and quality records, for a total of 480 evaluation epochs in each corresponding analysis table. The approach, docking, and line-securing phases additionally provide 360 near-field mooring records. Sixteen controlled fault events were introduced and independently labelled; no naturally occurring degradation was included in the fault-classification analysis. The native sensor system is multi-rate, whereas the statistical dataset uses synchronized evaluation epochs to support direct cross-modal comparison.

The completeness and phase coverage of the synchronized experimental database are documented in Table 7. The table reconciles run counts, common analysis epochs, labelled fault events, excluded runs, and additional phase-specific records, providing the denominator for every subsequent percentage and event count.

Table 7 confirms that all 40 runs and all 480 synchronized epochs were retained. The additional mooring, operator-event, and fault records provide phase-specific evidence without changing the common navigation-reference sample size. The zero-exclusion result simplifies interpretation but does not remove the need for stratification: only approach, docking, and line securing contain the 360 berth-relative rows, whereas all phases share the 480 navigation-reference epochs. Table 7 therefore prevents local-phase records from being incorrectly treated as a larger global-navigation sample.

### 6.2. Nominal Multisensor Fusion Accuracy

Across the 288 nominal evaluation epochs, the horizontal-position RMSE was 0.043 m, the MAE was 0.038 m, and the maximum horizontal error was 0.102 m. Heading RMSE and MAE were 0.176° and 0.140°, respectively, with a maximum error of 0.623°. Both horizontal and heading protection levels covered every nominal error sample. At run level, the mean horizontal RMSE was 0.041 m (bootstrap 95% CI: 0.036–0.046 m), and the mean heading RMSE was 0.168° (0.149–0.190°). These results characterize the achieved navigation accuracy and protection-level consistency of the multisensor system under the recorded nominal operating conditions.

**Figure 11:**
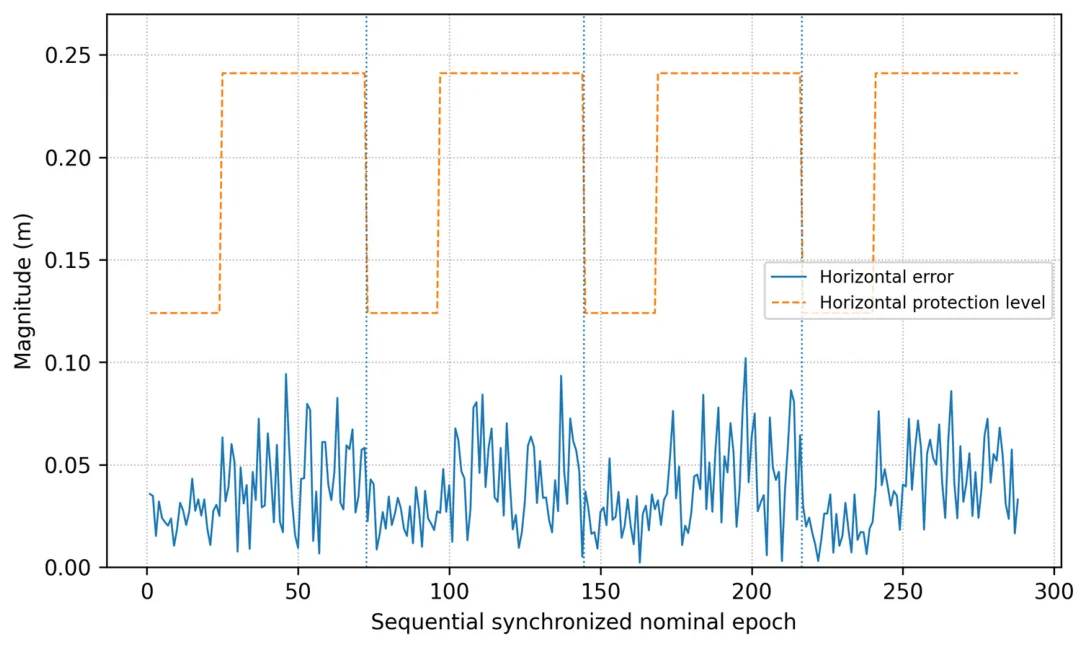
Horizontal-position error (solid blue curve) and corresponding phase-dependent horizontal protection level (dashed orange curve) across the 288 nominal synchronized epochs.

To evaluate nominal integrity rather than accuracy alone, Figure 11 compares the horizontal estimation error with the declared horizontal protection level at all 288 nominal synchronized epochs. Plotting both quantities on the same axis permits direct verification of empirical coverage and reveals changes in the phase-dependent integrity margin.

Figure 11 shows that every nominal horizontal error remains below its declared protection level. The step changes in the orange protection-level curve coincide with transitions among operational phases and reflect progressively tighter local-navigation requirements rather than deterioration of the nominal estimator. The separation between the curves represents the available integrity margin; its persistence across all nominal epochs supports the reported 100% nominal horizontal coverage.

### 6.3. Performance Under Single-Sensor Degradation

During 144 single-fault evaluation epochs, horizontal-position RMSE was 0.361 m and heading RMSE was 0.468°. Because all fault runs were acquired under adverse weather while nominal runs were calm or moderate, these values are descriptive condition-class summaries and are not interpreted as isolated causal fault effects. Horizontal protection-level coverage was 99.3%, corresponding to one protection-level non-envelopment epoch during GNSS dropout; heading protection-level coverage remained 100%. The uncovered epoch is explicitly classified as an integrity-bound failure because the 1.659 m horizontal error exceeded its contemporaneous protection level. It did not meet the operational HMI-event criterion because the error remained below the 2.0 m transit alert limit, but the failure of PL to bound the error is nevertheless a genuine integrity-monitoring defect. The most plausible mechanism is delayed covariance inflation during the transition from GNSS availability to dropout at the coarse temporal resolution of the synchronized diagnostic grid; insufficient high-rate internal logging prevents a unique attribution among delayed FDI response, covariance adaptation, and transient unmodelled bias. The mean run-level horizontal RMSE was 0.212 m (95% bootstrap CI: 0.085–0.409 m), reflecting substantial heterogeneity among fault types. The IMU-drift case produced the largest heading excursion (2.991°), while GNSS dropout and multipath dominated horizontal error. All 64 fault-active synchronized diagnostic records were identified, with no false-positive or missed-detection record among the 480 quality records. Mean event-log detection latency across the 16 events was 3.17 s (range 2.45–4.09 s), and mean controlled-recovery latency was 6.34 s (4.91–8.18 s). These latency values are quantized event-log measures at the synchronized diagnostic reporting resolution, not native 10–100 Hz algorithmic delays and not processor-cycle execution times.

**Figure 12:**
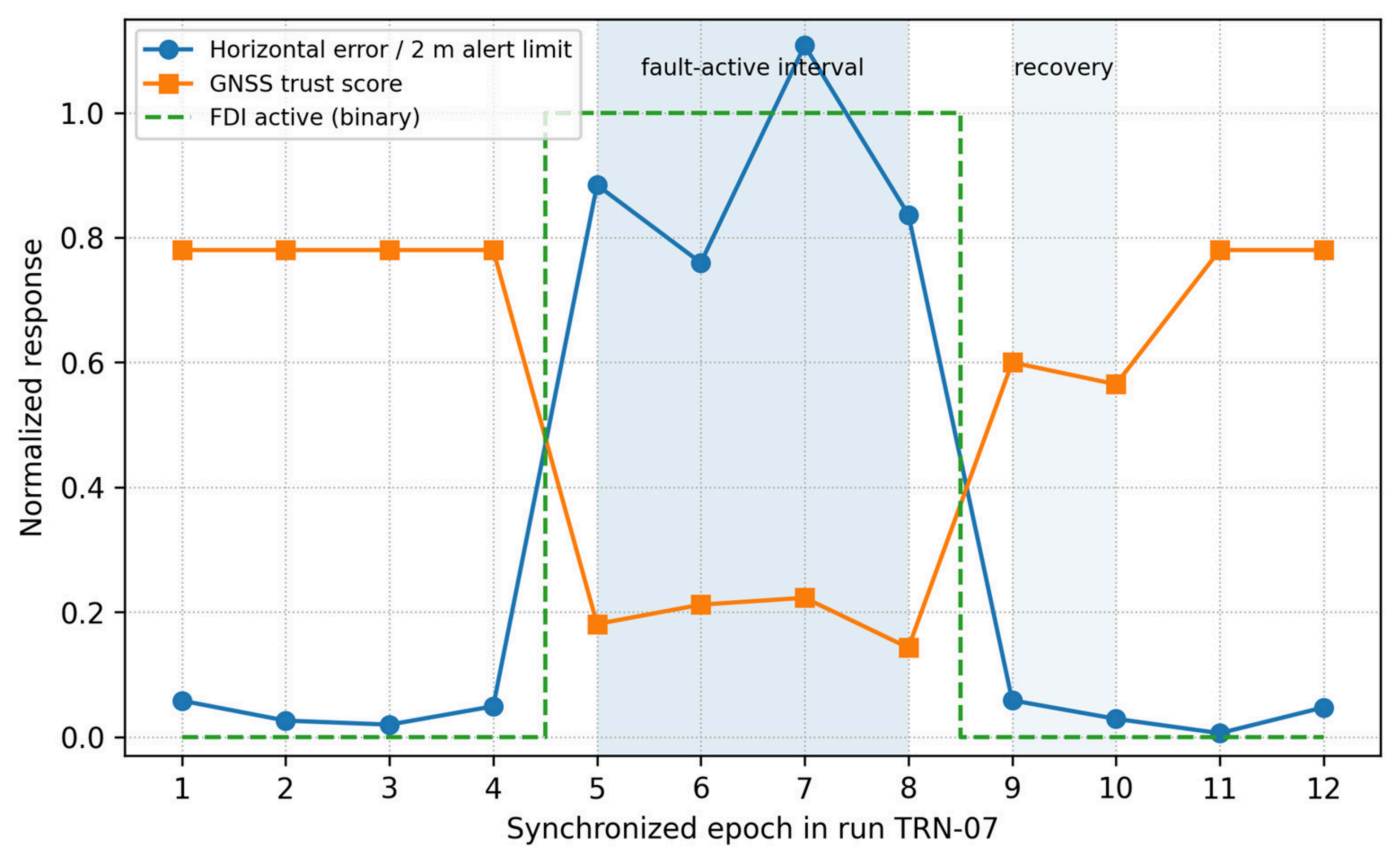
Dynamic response during the controlled GNSS multipath event in run TRN-07: normalized horizontal error (blue line with circular markers), GNSS trust score (orange line with square markers), binary FDI state (green dashed line), labelled fault-active interval (light-blue shading—the central region of the figure), and controlled-recovery interval (pale-blue shading—the right-hand region of the figure).

A representative high-severity GNSS multipath case is examined over time in Figure 12 so that the temporal relation among estimation error, trust adaptation, and the binary FDI decision can be assessed. The synchronized sequence includes a pre-fault baseline, a four-epoch labelled fault-active interval, and a controlled recovery interval.

During epochs 5–8 in Figure 12, normalized horizontal error increases sharply, GNSS trust decreases, and the FDI state becomes active. The three plotted signals therefore describe different but coupled functions: the blue curve quantifies navigation impact, the orange curve controls information weighting, and the green state records diagnostic classification. Epochs 9–10 form the controlled recovery interval, after which trust and error return toward their pre-fault range. This temporal correspondence is consistent with detection, down-weighting, and controlled re-entry, while causal attribution to any single algorithmic component still requires the replay-based ablation analyses identified in Section 8.

### 6.4. Performance Under Combined Faults and Time Misalignment

Forty-eight evaluation epochs belong to four combined-fault runs. Their horizontal-position RMSE was 0.598 m, with a maximum error of 2.320 m; heading RMSE was 0.342°. Horizontal and heading protection-level coverage remained 100% in the combined-fault subset. At run level, mean horizontal RMSE was 0.461 m, but the 95% bootstrap interval was wide (0.150–0.897 m) because only one combined-fault run was available per phase. Five consecutive epochs in the single combined-fault docking run DCK-10 were assigned an aborted outcome; the remaining 43 combined-fault epochs were labelled safe. This documents one conservative fail-safe sequence and must not be interpreted as five independent aborted trials. The five consecutive aborted decision epochs in DCK-10 are classified as one continuity and functional-availability interruption during final docking. They show conservative fail-safe behavior because unsafe autonomous action was withheld, but they do not demonstrate that the navigation/decision function remained continuously available. The exact interruption duration in seconds cannot be reconstructed from the 12-epoch synchronized analysis table, so its compliance with a docking-specific continuity-time requirement cannot be established from the present dataset.

**Figure 13:**
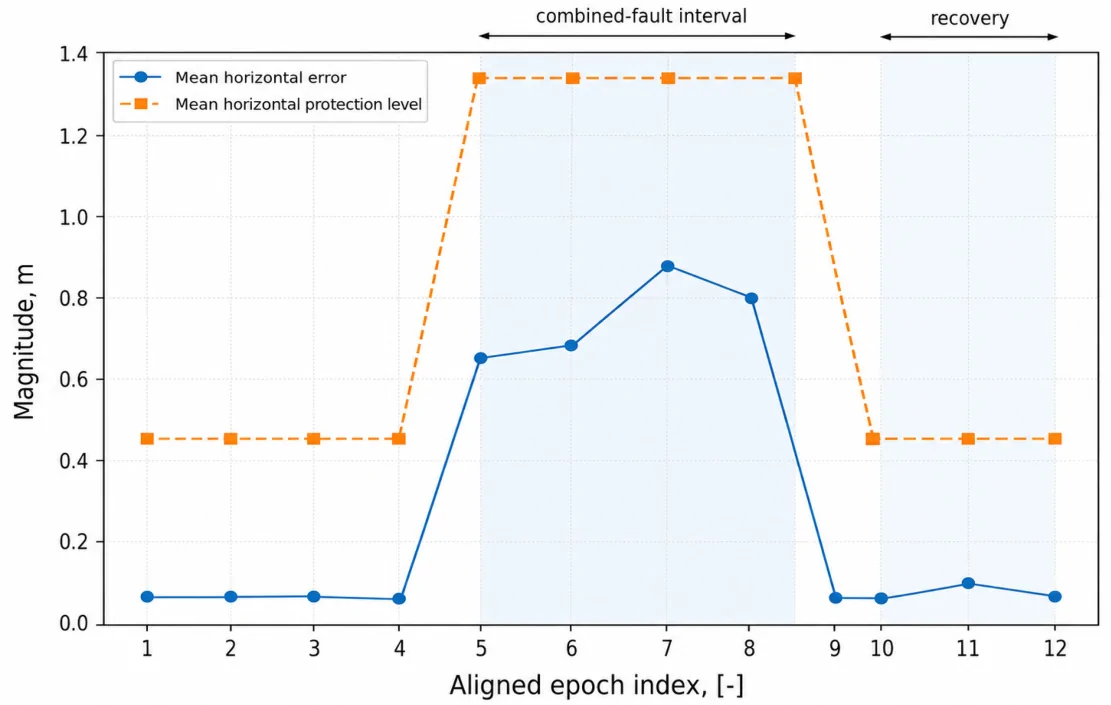
Aligned combined-fault response across four runs: mean horizontal error (solid blue line with circular markers) and mean horizontal protection level (dashed orange line with square markers); the light-blue-shaded central region marks the common combined-fault interval, whereas the pale-blue-shaded right-hand region marks the controlled-recovery interval.

The four combined-fault runs are aligned by the registered onset and recovery indices in Figure 13. Averaging the aligned sequences suppresses run-specific trajectory geometry and focuses the comparison on whether the protection level expands sufficiently during the common reduced-redundancy interval.

Figure 13 shows that the mean protection level expands at the onset of the aligned combined-fault interval and remains above the mean horizontal error throughout that interval. The blue error curve increases but does not intersect the orange integrity bound. After the labelled fault interval, both quantities contract toward their pre-fault values during recovery. This average supports conservative error bounding across the four aligned runs, but it must not be interpreted as uninterrupted functional continuity: DCK-10 contained a five-decision-epoch continuity/availability interruption that is masked by the four-run mean.

### 6.5. Integrity Calibration and Risk Prediction

The risk table contains 480 decision epochs. A total of 260 alerts were marked as correct in the adjudication field, and 220 non-alert states were marked as not assessable; no alert was labelled incorrect. Descriptively, mean composite risk increased from 0.331 under nominal sensing to 0.493 under single faults and 0.528 under combined faults, while mean integrity risk increased from 0.080 to 0.322 and 0.413. These between-condition values are not treated as causal fault effects because condition, weather class, and policy are partially confounded. Within the same *n* = 16 paired fault-run comparisons, however, mean composite risk increased from 0.412 during fault-inactive intervals to 0.680 during fault-active intervals (paired Wilcoxon *p* = 3.05 × 10^−5^), and mean integrity risk increased from 0.210 to 0.615 (*p* = 3.45 × 10^−4^). The log records 32 authority-mode switches, no unsafe-autonomy events or unnecessary takeovers, and five consecutive aborted decision epochs within DCK-10. A Brier score and expected calibration error were not calculated because a binary adverse-event outcome was not defined for every non-alert epoch. The interpretation therefore rests on within-run risk separation, alert adjudication, and observed safety outcomes rather than on full probability calibration [56,57].

**Figure 14:**
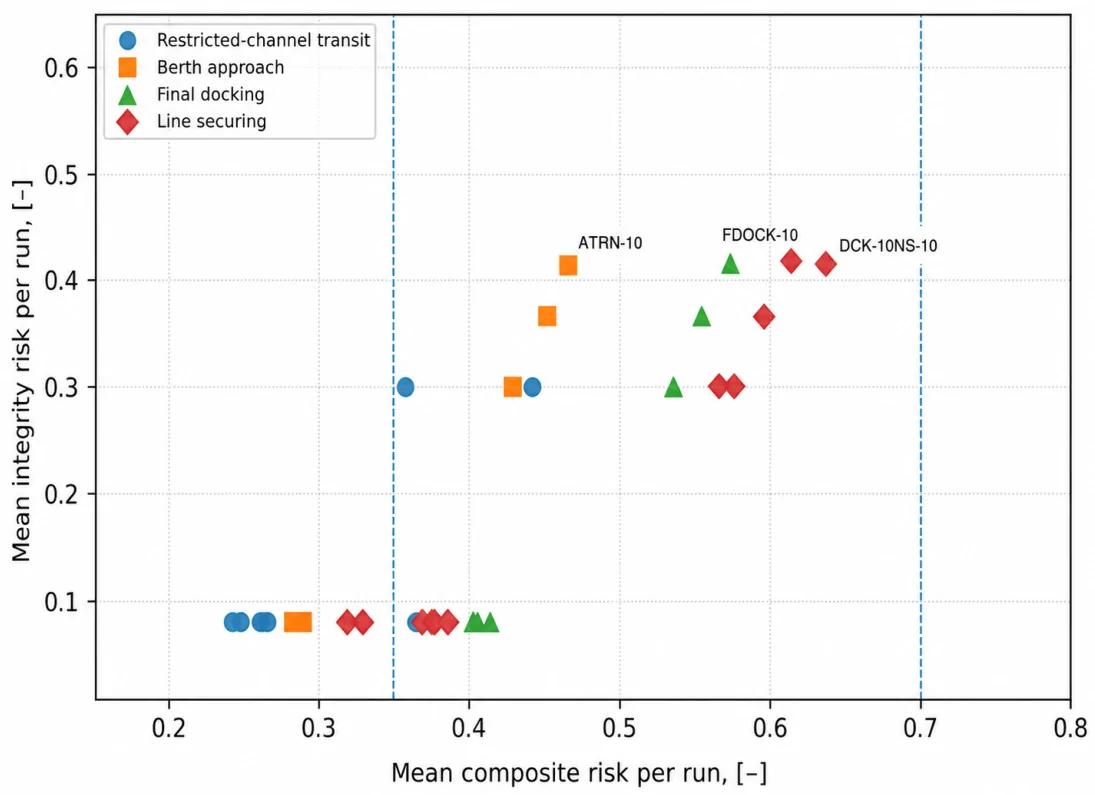
Run-level mean composite operational risk versus mean integrity risk by operational phase; marker color/shape denotes phase, dashed vertical lines show the advisory and protective composite-risk thresholds, and labels identify the four combined-fault runs.

To examine whether operational risk and measurement-integrity risk convey distinct information, Figure 14 plots their run-level means jointly and encodes operational phase by marker type and color. Combined-fault runs are labelled individually because they define the reduced-redundancy boundary of the experimental dataset.

Figure 14 separates nominal, single-fault, and combined-fault operation without requiring a binary adverse-event calibration target. Nominal runs cluster in the low-integrity-risk band, while combined-fault runs occupy the upper-right region. The vertical threshold lines act only on composite operational risk, whereas the vertical spread at similar composite risk demonstrates why integrity risk must remain an independent authority-allocation variable. The labelled combined-fault points also show that comparable integrity degradation can arise in different operational phases.

### 6.6. Human–Machine Authority Allocation

The recorded authority-state distribution comprised 210 recommendation epochs, 192 supervised-automatic epochs, 48 planned manual-reference epochs, and 30 protective-intervention epochs. The 48 manual-reference epochs correspond to four nominal runs assigned a manual authority policy and are not reactive takeovers. Sixteen operator-event records yielded a mean response latency of 4.83 s (median 4.85 s; range 2.6–7.4 s). Fourteen recommendations were accepted as issued, including two combined-fault events in which the accepted action was manual takeover; two recommendations were overridden without takeover. The records are explicitly classified as operational safety logging without a human-participant protocol. Accordingly, these results characterize system-level decision timing and do not support conclusions regarding workload, trust, cognition, or general operator behavior. The traffic-related decision variables are also consistent with AIS-based collision-avoidance analysis in busy waterways [58]. In the main-text terminology used here, “manual reference” always means a preplanned nominal comparator policy; only the two event-log takeover actions are counted as reactive manual takeovers.

### 6.7. Fault-Class Sensitivity and Computational Considerations

**Figure 15:**
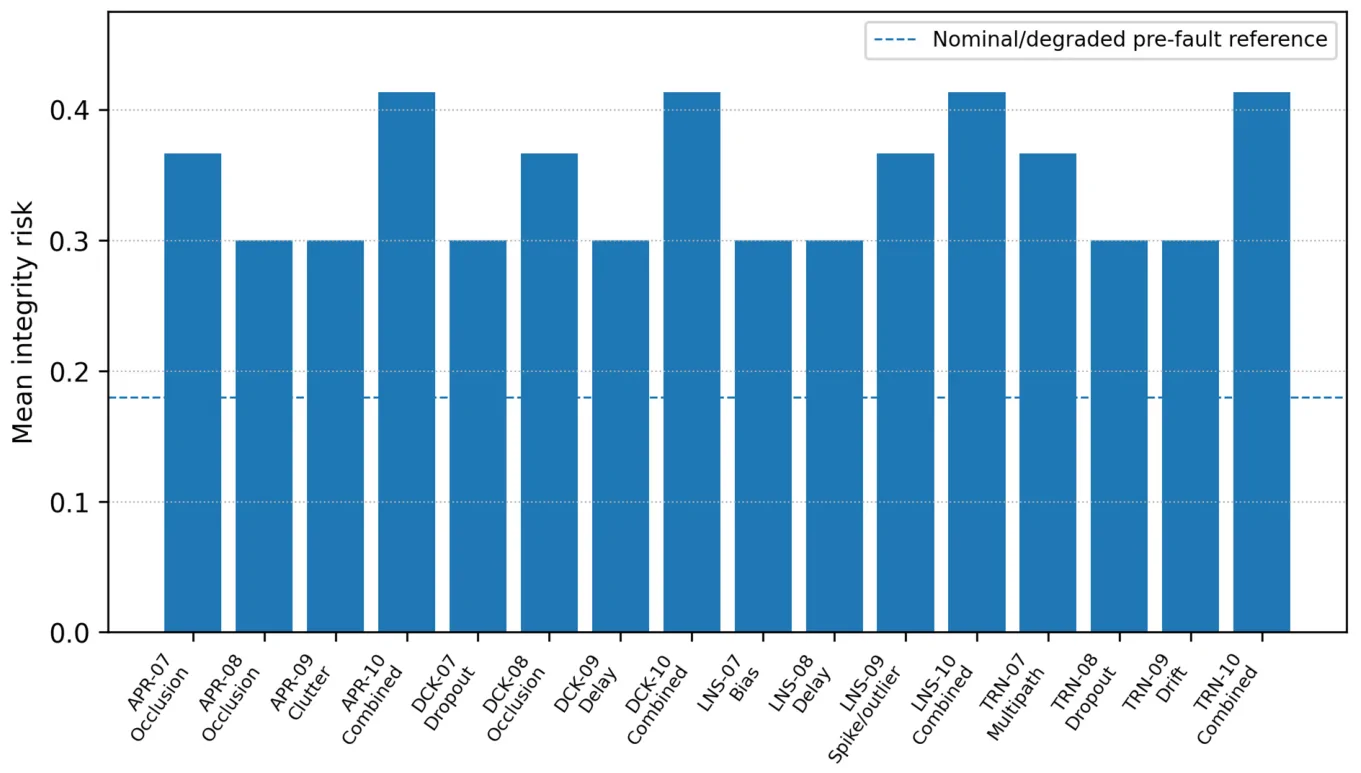
Mean integrity risk for the 16 controlled fault scenarios; bars correspond to individual labelled events and the horizontal dashed line denotes the nominal/degraded pre-fault reference level.

The experimental records do not contain independent estimator outputs for component-removal ablations; causal ablation claims are therefore not made. The available measurements support comparison across the 16 controlled fault events. Mean integrity risk took repeated values of 0.300, 0.367, and 0.413 for groups of scenarios. These repeated levels arise from the calibrated discrete integrity-state and rule-table mapping; they are derived decision outputs and do not imply identical raw sensor responses. The largest horizontal RMSE values occurred in the transit-phase GNSS multipath and combined GNSS degradation cases, whereas local line-load faults primarily affected tension estimation. Median recorded sensor-channel latency was 25.1 ms, the 95th percentile was 28.6 ms, and the largest delayed-channel value was 276.3 ms. End-to-end estimator execution time was not logged independently and is therefore not reported as a real-time computational benchmark. This discrete mapping reduces numerical granularity and creates tied integrity-risk values; therefore, differences within the same discrete tier cannot be interpreted from Figure 15 alone and must be examined using the continuous error, trust, innovation, and covariance variables.

Fault-class sensitivity is compared in Figure 15 using the mean integrity risk calculated for each of the 16 controlled events. The common dashed reference represents the nominal/degraded pre-fault level and allows the incremental effect of each fault family to be assessed on a consistent scale.

All labelled scenarios in Figure 15 lie above the pre-fault reference. Combined faults occupy the highest integrity-risk tier; multipath, severe occlusion, and spike/outlier events form an intermediate tier; and several delay, bias, drift, clutter, and dropout cases remain lower but elevated. The repeated bar heights reflect the discrete risk-state mapping described above and should be interpreted together with continuous error, trust, and covariance measurements rather than as identical physical responses.

The evidence supporting each research hypothesis is consolidated in Table 8. The table cross-references the baseline or fault-inactive condition, the degraded response, the quantitative interpretation, and the final level of support, making explicit where the evidence is direct, descriptive, or incomplete.

Table 8 indicates that H2 has the strongest direct support, while H1, H3, H4, and H5 are supported with explicitly stated qualifications. This graded assessment avoids overstating causal attribution beyond the measurements and configurations contained in the database. H2 receives direct event-level support because independent fault labels are available, whereas H1, H3, H4, and H5 retain qualifications arising from channel specificity, line-load sensitivity, incomplete probability calibration, and the absence of a matched fixed-policy replay. This hierarchy aligns the final claims with the actual inferential strength of the experiment.

## 7. Discussion

### 7.1. Interpretation of the Main Findings

The principal finding is not invariant accuracy under degradation but rather an integrity response whose limitations can be identified explicitly. Nominal horizontal-position and heading RMSE were 0.043 m and 0.176°. The descriptive condition-class values were 0.361 m and 0.468° for single faults and 0.598 m and 0.342° for combined faults. These differences cannot be assigned causally to fault injection because all degraded runs occurred under adverse weather. An unstratified paired comparison across all 16 fault runs also did not show a uniform active-versus-inactive increase in horizontal or heading error (Wilcoxon *p* = 0.744 and *p* = 0.900), because several local-perception and line-load faults do not directly observe the global navigation components. Horizontal PL coverage was 99.3% for single-fault epochs and 100% for combined-fault epochs; heading coverage was 100%. The single uncovered GNSS-dropout epoch is now reported as an integrity-bound failure: its 1.659 m error exceeded PL. Because the error remained below the 2.0 m transit alert limit, it was not an operational HMI-event, but the non-envelopment itself is a defect that requires correction before deployment. H1 is therefore only partially supported.

H2 has the strongest direct support. All 64 independently labelled fault-active quality records were detected, and all 416 fault-inactive records were classified without a false alarm. These observations are synchronized diagnostic records nested within 16 fault events; they are not 480 independent trials or an external validation set. Mean trust decreased from 0.863 in fault-inactive records to 0.198 during active faults, while mean covariance scale increased from 1.16 to 5.57. The reported 3.17 s detection latency and 6.34 s recovery latency are event-log values at the synchronized reporting resolution. They must not be interpreted as native-rate algorithmic or processor delays. Broader performance estimates require additional vessels, sites, fault repetitions, and high-rate diagnostic logging.

The phase-specific results support H3 with an important qualification. Berth-relative distance RMSE was 0.032 m during approach, 0.018 m during final docking, and 0.012 m during line securing; relative-angle RMSE remained within 0.375–0.410°. These values show that centimeter-scale local pose estimation was maintained across the recorded local phases, but the cross-phase decrease cannot be attributed causally to phase adaptation because geometry, maneuvering state, and active observables also change. Line-tension estimation was substantially more sensitive: RMSE increased from 0.743 kN under nominal sensing to 4.339 kN for single faults and 3.349 kN for combined faults, with a 24.68 kN maximum error during the injected spike. The evidence therefore supports local pose continuity and identifies line-load monitoring as the principal channel requiring stronger redundancy and transient rejection.

H4 receives stronger support from the paired within-run analysis than from the confounded condition classes. Across n = 16 paired fault-run comparisons, fault-active intervals had higher mean composite risk than fault-inactive intervals (0.680 versus 0.412; paired Wilcoxon *p* = 3.05 × 10^−5^) and higher mean integrity risk (0.615 versus 0.210; *p* = 3.45 × 10^−4^). All 260 issued alerts were marked correct in the experimental log. DCK-10 nevertheless contained one five-decision-epoch continuity/availability interruption. No unsafe-autonomy or adjudicated unnecessary-takeover event was recorded. H4 remains incompletely calibrated because binary adverse-event outcomes are unavailable for all non-alert epochs. H5 is treated only as an exploratory description of the recorded adaptive-policy sequence. Without matched replay of static shared-control or full-autonomy policies on the same logged measurements, no causal claim of superiority or takeover reduction is made.

Table 8 summarizes the graded hypothesis assessment. H1 is only partially supported because channel-specific degradation was generally bounded but one GNSS-dropout epoch produced protection-level non-envelopment. H2 is supported directly by independent fault labels. H3 is supported for recorded local pose continuity and only partially for line-load estimation. H4 is supported by significant within-run risk separation and alert adjudication, while formal probability calibration remains unavailable. H5 is retained only as an exploratory description of the recorded adaptive decision sequence; a matched fixed-policy replay is required before any claim of comparative superiority.

### 7.2. Comparison with Previous Sensor-Fusion Approaches

The local-perception literature has established the feasibility of RTK/IMU/LiDAR berthing perception and multi-LiDAR/millimeter-wave radar fusion [1,2], together with shipborne 3D-LiDAR berth-state estimation [3,4]. The present results are consistent with that body of work: centimeter-scale berth-distance accuracy and sub-degree relative-angle accuracy were maintained across approach, docking, and line-securing phases. Direct claims of numerical superiority are not appropriate because vessel geometry, reference systems, berth layout, environmental conditions, and reporting metrics differ among studies. The distinctive contribution here is the connection of local pose estimates to a continuous global navigation state, per-channel quality diagnostics, protection levels, and authority allocation. Risk assessments of maritime autonomous surface ships similarly emphasize that operational functions, failure modes, and remote-control arrangements must be evaluated jointly [59].

Radar–AIS and AIS–visual fusion studies have primarily emphasized integrated motion perception [5,6], while simulation-based work has isolated data-association uncertainty as a separate validation problem [7]. In the proposed architecture, these modalities retain that perception role but are also treated as integrity-bearing measurements whose latency, confidence, and cross-sensor residuals modify the fused covariance and operational risk. This distinction is relevant in confined waters, where a correctly associated target does not by itself guarantee that the own-ship state or berth-relative solution is sufficiently trustworthy for autonomous action. The observed traffic and berth constraints should additionally be interpreted within phase-specific ship safety domains rather than by a single invariant clearance boundary [60].

The navigation-integrity component extends robust GNSS/INS approaches [8,9] by including phase-dependent exteroceptive and near-field observations. The empirical protection-level behavior confirms the value of separating accuracy from integrity: the largest errors occurred under GNSS-related degradation, yet the decision layer remained informed by an explicit bound rather than only by the point estimate. Relative to fault-tolerant integrated-navigation fusion based on fuzzy inference [10], the present framework maintains an auditable chain from quality indicators and sensor mode to covariance scale, protection level, risk, and final authority mode.

The human–machine literature argues for cooperative and supervisory forms of maritime autonomy rather than a permanently fixed allocation of control [11,12]. The current experiment operationalizes that principle using measured sensor integrity as an explicit authority variable. This does not replace human-factor validation, but it provides a technical basis for explaining why the same geometric situation can lead to autonomous action when integrity is high and to recommendation, protective intervention, or takeover when the sensing solution is uncertain.

### 7.3. Implications for Sensor-System Design

The results indicate that useful redundancy must be functional rather than merely numerical. Duplicate sensors that share the same obstruction, clock, power supply, or environmental sensitivity do not provide the same resilience as heterogeneous channels. The experimental architecture combines absolute positioning, inertial propagation, geometric perception, cooperative traffic data, short-range distance, actuation response, environmental disturbances, and line loads. This diversity allowed the estimator to retain an observable subset when an individual modality was degraded.

Time architecture is a first-order design variable. The sensor suite spans 1–100 Hz, and controlled delay and timestamp-offset faults produced identifiable changes in innovation consistency and trust. A deployable system should therefore use a traceable common clock, preserve acquisition timestamps rather than logger-arrival times, record latency estimates with each observation, and test for time offsets before spatial residuals are interpreted as physical sensor error. Re-entry logic should also include timing stability, because a recovered measurement with an unresolved delay can be more hazardous than an explicitly unavailable channel.

Sensor placement should be optimized by phase and failure geometry. Global-navigation antennas and inertial units require lever-arm and alignment calibration; radar, LiDAR, and cameras require overlapping berth and traffic coverage; ultrasonic channels require blind-zone and specular-reflection analysis; and line-tension sensors require overload margin, transient filtering, and preferably independent verification. The elevated tension error during spike and bias tests demonstrates that line-load monitoring should not rely on covariance adaptation alone. A physically based rate limit, cross-line equilibrium test, and actuator/load consistency residual should be retained as separate protection barriers.

Finally, alert limits and quality-to-covariance mappings must be phase dependent. A covariance acceptable for channel transit may be unacceptable during final docking, while the sensor that dominates transit accuracy may be less informative than berth-relative LiDAR or ultrasonic ranging near contact. The recorded decrease in distance RMSE toward final docking supports this reweighting principle. Calibration should therefore deliver not a single nominal covariance matrix, but a family of bounded models indexed by sensor mode, environment, operational phase, and recovery state.

### 7.4. Implications for Autonomous and Human-Supervised Operation

For autonomous operation, the experiment demonstrates the importance of separating navigation risk from information integrity. High geometric risk with high integrity can justify rapid protective action; low apparent risk with poor integrity requires caution because the low-risk estimate may itself be unreliable. Protective intervention occurred only in fault-affected epochs. The five aborted outcomes were consecutive decision epochs within one combined-fault docking run and represent one intentional fail-safe sequence, not five independent failures. Planned manual-reference runs are analyzed separately from event-triggered operator actions. From a human–automation perspective, the policy focuses on appropriate reliance rather than maximizing either automation use or manual intervention [61].

Manual takeover should not be treated as a binary indicator of system failure. The 48 manual-reference decision epochs were planned nominal comparators. In the operator-event log, fourteen recommendations were accepted as issued; two of those accepted recommendations called for manual takeover during combined faults, while two other recommendations were overridden without takeover. Mean response latency was 4.83 s. These observations characterize the recorded decision-support loop only; they do not constitute a general model of human response because the log contains two research operators and sixteen events.

An operational interface should expose the causal chain behind each recommendation: affected sensor, detected fault mode, trust reduction, protection-level consequence, dominant risk term, and expected recovery condition. Such explanations allow the operator to distinguish a maneuvering hazard from a sensing-integrity hazard and to select an appropriate response. They also provide traceability for post-event analysis and threshold refinement.

The reported absence of unsafe-autonomy and unnecessary-takeover events is encouraging but does not constitute certification evidence. Certification would require formal hazard analysis, independent verification of software and hardware, common-cause failure assessment, cybersecurity testing, environmental qualification, and a substantially broader operational design domain. The present contribution is an experimentally evaluated sensing and decision-support architecture that can provide quantitative evidence for those subsequent assurance processes.

### 7.5. Generalizability and Transfer Conditions

Several architectural elements are transferable without changing their conceptual form: asynchronous event-driven fusion, the multidimensional sensor-quality vector, innovation and cross-sensor FDI, controlled recovery, component-specific protection levels, and joint risk-integrity authority logic. These elements describe relationships among measurements and decisions rather than a particular vessel or port.

The numerical parameters are not directly transferable. A new installation requires recalibration of sensor extrinsics, lever arms, time offsets, nominal measurement covariances, process-noise levels, innovation thresholds, persistence counters, trust mappings, bias bounds, protection-level multipliers, dynamic-model parameters, hazard consequence weights, alert limits, and minimum authority dwell times. Berth geometry and vessel maneuvering characteristics are especially important for contact-energy and line-load risk.

A defensible transfer process should proceed hierarchically. First, sensor and timing calibration should be repeated under static and controlled dynamic conditions. Second, nominal and single-fault trials should verify accuracy, detection, and protection-level coverage on the new vessel and berth. Third, combined faults and environmental extremes should be introduced after the single-channel mechanisms are understood. Finally, multi-site and multi-vessel evaluation should test whether normalized quality and risk features can share common parameter ranges or require platform-specific models.

The present campaign covers the calm, moderate, and adverse environmental-condition classes represented in the database, but it does not span all visibility, precipitation, sea-state, traffic, and infrastructure combinations. Generalization should therefore be stated in terms of the validated operational envelope. Extension beyond that envelope requires explicit detection of out-of-distribution conditions and a conservative reduction of autonomy until new evidence is accumulated.

## 8. Limitations

The first limitation concerns the temporal resolution of the archived analysis layer rather than the native estimator architecture. The twelve synchronized epochs per run are common posterior reporting points extracted from an asynchronous estimator that processes native 1–100 Hz sensor events. They support state, error, and integrity comparisons at those common epochs, but they do not retain enough packet-level detail to reconstruct sub-second transients or native-rate FDI and processing latency.

The study has several material limitations. First, the statistical dataset contains twelve synchronized evaluation epochs per run. Native acquisition is multi-rate, but packet-level loss, sub-second transients, and native-rate diagnostic delay cannot be reconstructed fully from the analysis table. Second, individual fault mechanisms and severity levels have limited replication, so fault-specific estimates characterize this campaign rather than population failure probabilities. Third, all injected-fault runs were acquired in adverse weather, while nominal runs were calm or moderate and authority policies also differed among nominal runs. Between-condition comparisons are therefore descriptive and partially confounded. Fourth, simultaneous degradation of heterogeneous sensors can create correlated residuals and common-cause loss of observability; the present FDI architecture mitigates this through cross-sensor consistency checks and active-set reconfiguration but cannot guarantee isolation when several channels fail coherently. Slowly developing or subtle biases are also harder to detect than abrupt faults. Fifth, the synchronized diagnostic export does not permit separation of native algorithmic latency from reporting-grid latency. Sixth, exact modality-specific trust coefficients, persistence thresholds, integrity multipliers, unresolved-fault weights, and policy weights are retained in the processing configuration but are not recoverable from the synchronized analysis export supplied for this manuscript; independent numerical reproduction therefore requires release of that configuration. Seventh, the common solution-source label prevents component-wise estimator ablations, and no matched fixed-policy counterfactual replay is available for H5. Eighth, the risk records lack a binary adverse-event outcome for every non-alert epoch, preventing complete Brier-score and expected-calibration-error analysis. Ninth, hydrodynamic bank/shore effects are represented only through disturbance-state uncertainty; a dedicated high-fidelity bank-effect interaction model was not identified from the present campaign. The architecture can inflate process uncertainty when shore interaction grows, but it does not remove hydrodynamic-model error. Tenth, small fast-moving or non-cooperative obstacles can remain weakly observed between radar, LiDAR, camera, AIS, and ultrasonic updates. Multimodal fusion reduces single-sensor exposure, but target appearance/disappearance and association uncertainty remain failure modes, especially under occlusion or clutter. Eleventh, uncertainty propagation uses covariance, bounded bias/fault terms, and short-horizon stochastic hazard propagation; strongly non-Gaussian correlated failures can therefore be underrepresented. Twelfth, the five aborted DCK-10 decision epochs constitute a continuity and functional-availability interruption. They were fail-safe because unsafe autonomy was withheld, not because the function remained continuously available; the synchronized dataset is insufficient to verify whether the interruption duration satisfies a docking-specific continuity requirement. Thirteenth, operator events are operational safety logs rather than a participant-centered human-factors study. The authority policy formalizes transfer conditions, hysteresis, explanation codes, and minimum dwell times, but it does not resolve the mismatch between the operator’s mental model and the automation’s internal risk/integrity model. Workload, trust calibration, mode awareness, transfer-of-control comprehension, and interface usability require dedicated human-participant testing. Fourteenth, the campaign covers one vessel, one sensor layout, one confined-water geometry represented in local coordinates, and the recorded environmental envelope; site geography and bathymetry are withheld from the analytical release. Fifteenth, the controlled fault set excludes several common-cause power-supply and network failures, spoofing and cybersecurity attacks, and coordinated sensor-deception failures. Sixteenth, end-to-end estimator execution time and memory use were not independently profiled. Finally, formal hazard analysis, independent software assurance, environmental qualification, and certification testing were outside the scope [62,63].

For reproducibility, the same archival limitation applies to the exact inertial white-noise spectral densities and accelerometer/gyroscope bias random-walk intensities: these quantities belong to the processing-configuration material and cannot be reconstructed uniquely from the synchronized analysis export.

For camera and LiDAR channels, scenario dependence also arises from the perception front end itself: feature extraction, segmentation, tracking, or association can return no valid observation or a biased feature product even when the physical sensor remains operational. The quality/confidence logic reduces the influence of such products but does not guarantee detection of every coherent perception error.

## 9. Conclusions

This study developed and experimentally evaluated a risk-aware fault-tolerant multisensor architecture for the continuous sequence of restricted-channel transit, berth approach, final docking, and mooring-line securing. The architecture integrates 24 heterogeneous sensing and feedback channels through asynchronous quality-aware error-state fusion, explicit fault detection and isolation, controlled sensor reconfiguration, phase-dependent protection levels, operational risk propagation, and human–machine authority allocation.

The five-day experimental campaign comprised 40 completed runs, 480 synchronized evaluation epochs, and 16 independently labelled controlled fault events. Under nominal sensing, horizontal-position and heading RMSE were 0.043 m and 0.176°. Under single faults, the corresponding descriptive values were 0.361 m and 0.468°; under combined faults, they were 0.598 m and 0.342°. These condition-class differences are descriptive because weather class and fault condition were confounded. Horizontal PL coverage was 100% in nominal conditions, 99.3% under single faults, and 100% under combined faults; heading coverage was 100% throughout. The 99.3% result includes one GNSS-dropout epoch in which horizontal error exceeded PL. This epoch is explicitly classified as an integrity-bound failure, although it was not an HMI-event because the 1.659 m error remained below the 2.0 m transit alert limit.

All 64 fault-active diagnostic records were identified without a false-positive or missed-detection record in the completed campaign. Mean event-log detection and controlled-recovery latencies were 3.17 s and 6.34 s. Mean sensor trust decreased from 0.863 in fault-inactive records to 0.198 during active faults, while the mean covariance scale increased from 1.16 to 5.57. Berth-relative distance RMSE remained between 0.012 and 0.032 m across the local phases, whereas line-tension estimation showed substantially greater sensitivity to bias and spike faults.

The decision layer exhibited a traceable response to active sensing degradation. Across n = 16 paired fault-run comparisons, mean composite risk increased from 0.412 in fault-inactive intervals to 0.680 in fault-active intervals (paired Wilcoxon *p* = 3.05 × 10^−5^), while mean integrity risk increased from 0.210 to 0.615 (*p* = 3.45 × 10^−4^). The system recorded 32 authority-mode switches and 260 alerts marked correct. No unsafe-autonomy events or adjudicated unnecessary takeovers were logged. One combined-fault docking run generated five consecutive aborted decision epochs, which are classified as one continuity/functional-availability interruption and one conservative fail-safe sequence. These observations do not establish superiority of the adaptive authority policy because no matched fixed-policy counterfactual replay was available.

The hypotheses received different levels of support. H1 was only partially supported because channel-specific degradation was generally integrity bounded but one GNSS-dropout epoch produced PL non-envelopment. H2 was directly supported by independent fault labels. H3 was supported for local pose continuity but only partially for line-load sensing. H4 was supported by significant within-run risk separation but not by full probability calibration. H5 is retained as an exploratory description of the observed adaptive authority sequence; no causal comparison with a fixed policy is claimed without matched replay.

The next validation stage should process the full-rate raw streams, execute fixed-covariance and component-removal replays, expand repeated fault trials and environmental coverage, and test the architecture on additional vessels and berths. Particular priority should be given to redundant line-load protection, common-cause timing and network failures, out-of-distribution detection, and an independent comparison of adaptive and static authority policies. These steps will determine the transferability of the architecture and provide the evidence required for formal maritime safety assurance.

## Figures and Tables

**Table 1:** Positioning of representative approaches relative to the proposed architecture.

Study Group	Sensor Modalities	Quality/FDI	Integrity	Risk Propagation	Human–Machine Layer	Principal Limitation Relative to This Study
RTK/IMU/LiDAR berthing perception [1]	RTK-GNSS, IMU, LiDAR	Partial	No explicit PL	No	No	Focuses on berth-perception performance; end-to-end integrity-conditioned authority allocation is outside its scope.
Multi-LiDAR/MMW radar berthing [2]	LiDAR, MMW radar	Synchronization oriented	No	No	No	Focuses on relative berth-state estimation and sensor synchronization; navigation-integrity and authority-allocation layers are outside its scope.
Shipborne 3D LiDAR berthing [3,4]	3D LiDAR	Limited	No	No	No	Provides high-resolution local berth geometry from 3D LiDAR; heterogeneous integrity redundancy is not a stated objective.
Radar/AIS/camera motion perception [5,6]	Radar, AIS, cameras	Association robustness	No	Partial	Operator surveillance	Addresses traffic perception and data association; full own-ship/mooring integrity monitoring is outside its stated scope.
Robust GNSS/INS integrity [8,9]	GNSS, INS	Yes	Yes	No	No	Provides a strong GNSS/INS integrity core; phase-dependent maritime exteroceptive integration is not within the stated study scope.
Fault-tolerant federated navigation [10]	Integrated navigation subsystems	Yes	Partial	No	No	Addresses fault-tolerant integrated-navigation weighting; propagation into operational risk and authority allocation is not its stated objective.
Human–machine cooperative navigation [11,12]	System-level information	Indirect	Indirect	Yes	Yes	Addresses cooperative/supervisory authority concepts; explicit coupling to per-sensor integrity metrics is not the primary methodological focus.
Present experimental framework	24 heterogeneous channels	Yes	Phase-specific PL/HMI	Yes	Joint risk–integrity policy	Integrated experimental chain from quality/FDI to integrity, risk, and authority; external validity across multiple vessels and sites remains untested.

**Table 2:** Heterogeneous sensor architecture used in the experimental campaign.

Group	Modality and Channel Count	Primary Observables	Nominal Rate	Operational Role
Navigation core	Septentrio AsteRx-U3 RTK-GNSS (1); VectorNav VN-300 IMU (1); KVH P-1775 gyrocompass (1)	Absolute position/velocity; specific force; angular rate; heading	20/100/100 Hz	High-rate global state and short-term continuity
External perception	Navtech CTS350-X radar (1); Ouster OS1-64 LiDAR (1); Basler ace 2 cameras (2); SRT Class A AIS (1)	Range/bearing; point cloud; image features; cooperative target state	10/10/20/1 Hz	Traffic, shoreline, berth, and obstacle perception
Near field	Pepperl + Fuchs UC6000 ultrasonic sensors (4)	Distance to berth or obstacle	20 Hz	Final-approach and docking clearance
Actuation feedback	TC-100 thrust channels (2); Novotechnik RFC-4800 rudder (1); HBM T40B shaft-speed channels (2)	Commanded/achieved propulsion and steering response	20 Hz	Dynamic-model input and actuator diagnostics
Mooring loads	HBM U10M line-tension load cells (4)	Individual mooring-line tension	20 Hz	Overload prevention and line-securing state
Environment	Gill WindSonic (1); Nortek Aquadopp (1)	Disturbance speed and direction	1 Hz	Risk and model adaptation
Derived perception	MSSF v1.2 fused-object track (1)	Associated target state and confidence	10 Hz	Common object representation for the decision layer

**Table 3:** Research hypotheses, primary comparisons, and experiment-level acceptance criteria.

ID	Hypothesis	Primary Comparison	Acceptance Evidence
H1	Sensor degradation produces channel-specific accuracy loss, while protection levels remain conservative.	Nominal summaries plus fault-active/fault-inactive intervals, stratified by affected state	Position/heading error, PL coverage, alert-limit exceedance, trust and covariance response
H2	FDI identifies labelled fault-active records and enforces controlled recovery.	Independent fault labels vs. synchronized diagnostic records	TP/TN/FP/FN, event-log detection latency, recovery latency, continuity
H3	Phase-adaptive sensor relevance preserves local-state estimation during approach and mooring.	Transit, approach, docking, and line-securing phases	Berth-distance/angle error, line-tension error, phase-specific availability
H4	Fault-active intervals increase composite and integrity risk within the same run.	Paired active vs. inactive intervals in the 16 fault runs	Paired Wilcoxon test, alert adjudication, aborted and unsafe outcomes
H5	The recorded risk–integrity policy exhibits conservative authority escalation in the observed campaign.	Recorded adaptive-policy authority states and operator-event log; no matched static-policy replay	Mode switches, unsafe-autonomy events, adjudicated takeovers, response latency, and explicit counterfactual limitation

**Table 4:** Completed experimental program by operational phase and sensing condition.

Scenario Group	Phase	Nominal Runs	Single-Fault Runs	Combined-Fault Runs	Principal Labelled Degradations
TRN	Restricted-channel transit	6	3	1	GNSS multipath, dropout, IMU drift, combined GNSS bias/delay
APR	Berth approach	6	3	1	LiDAR/camera occlusion, radar clutter, combined LiDAR/range bias
DCK	Final docking	6	3	1	Ultrasonic dropout, camera occlusion, radar delay, combined proximity/vision loss
LNS	Line securing	6	3	1	Tension bias, thrust delay, tension spike, combined tension/vision degradation
Total	All phases	24	12	4	40 runs and 16 labelled fault events

**Table 5:** Operational configurations and fault-study variants encoded in the experimental dataset.

Code	Experimental Configuration	Role in the Present Analysis
B1	Manual authority policy; nominal condition	Planned manual-reference comparator, not an event-triggered takeover
B2	Full-autonomy authority policy; nominal condition	Autonomy reference under nominal sensing
B3	Static shared-control authority policy; nominal condition	Fixed shared-authority reference
B4	Risk–integrity adaptive authority policy; nominal condition	Adaptive-policy reference under moderate envelope
B5	Full-autonomy authority policy; nominal condition	Additional nominal full-autonomy condition
P	Risk–integrity adaptive policy under nominal and selected single faults	Full proposed decision chain
A1	Risk–integrity adaptive policy with dropout/occlusion/delay faults	Fault-family study
A2	Risk–integrity adaptive policy with drift/clutter/delay/spike faults	Fault-family study
A3	Risk–integrity adaptive policy with combined faults	Reduced-redundancy study

**Table 6:** Evaluation metrics used for accuracy, integrity, risk, and decision-support analysis.

Category	Metric	Unit/Definition	Reporting Requirement
State accuracy	Position, heading, velocity RMSE/MAE	m, deg, m/s	RMSE, MAE, maximum error, and run-level bootstrap CI
Relative docking	Berth-distance and relative-angle error	m, deg	By phase and sensing condition
Fault management	Detection probability, false alarm, missed detection	%	Per fault type and severity
Fault management	Detection and recovery latency	s	Mean, range, and event count at synchronized diagnostic resolution
Integrity	Protection level and HMI probability	m/deg; probability	Coverage and calibration
Continuity	Valid-solution availability and interruption duration	%; s	Per scenario
Risk	Violation probability, alert precision/recall, Brier score, ECE	dimensionless	Risk separation and alert adjudication; Brier/ECE only when binary outcomes are available
Decision support	Unsafe autonomy, unnecessary takeover, switching rate	count/rate	By policy
Computational	Recorded sensor-channel latency	ms; acquisition/communication timing	Median, 95th percentile, and maximum; not estimator execution time

**Table 7:** Experimental dataset composition and synchronized-analysis completeness.

Phase/Scenario	Runs	Nominal/Single/Combined	Synchronized Epochs	Fault Events	Excluded Runs	Additional Phase-Specific Rows
Restricted-channel transit	10	6/3/1	120	4	0	AIS/radar and navigation-risk fields
Berth approach	10	6/3/1	120	4	0	120 berth-relative measurement rows
Final docking	10	6/3/1	120	4	0	120 berth-relative measurement rows
Line securing	10	6/3/1	120	4	0	120 berth-relative and line-tension rows
Total	40	24/12/4	480 per common analysis sheet	16	0	360 mooring rows; 480 reference rows

**Table 8:** Consolidated result summary and hypothesis assessment.

Hypothesis	Primary Evidence	Reference or Fault-Inactive Condition	Degraded/Proposed Response	Quantitative Interpretation	Assessment
H1	State error and protection-level response	Nominal: 0.043 m/0.176°; PL coverage 100%/100%	Single: 0.361 m/0.468°, PL 99.3%/100%; combined: 0.598 m/0.342°, PL 100%/100%	Aggregate comparisons are confounded; unstratified active/inactive error tests *p* = 0.744 and *p* = 0.900	Partially supported: channel-specific degradation was observed, but one single-fault GNSS-dropout epoch produced PL non-envelopment.
H2	FDI classification, trust, covariance, latency	416 fault-inactive records; trust 0.863; covariance scale 1.16	64 TP; 0 FN; 0 FP; trust 0.198; scale 5.57; detection/recovery 3.17/6.34 s	100% sensitivity and specificity within the 16 labelled events at synchronized diagnostic resolution	Supported within the completed campaign
H3	Berth-relative pose and line-tension error	Nominal: distance RMSE 0.019 m; angle 0.366 deg; tension 0.743 kN	Single: 0.028 m; 0.423 deg; 4.339 kN. Combined: 0.024 m; 0.498 deg; 3.349 kN.	Local pose remained stable; line-load estimation was fault sensitive	Partially supported; local pose maintained, line-load channel fault sensitive
H4	Paired within-run risk response	Fault-inactive: composite/integrity risk 0.412/0.210	Fault-active: 0.680/0.615; 260 alerts marked correct; one run with five aborted epochs	Paired Wilcoxon *p* = 3.05 × 10^−5^ and *p* = 3.45 × 10^−4^	Supported for within-run risk response; probability calibration pending
H5	Recorded adaptive authority response (exploratory H5)	48 planned manual-reference epochs; nominal runs had 0 switches	Fault epochs: 114 recommendation, 48 supervised automatic, 30 protective; 32 switches; two actual takeovers	0 unsafe autonomy; 0 unnecessary takeover; 14 accepted, 2 overridden; mean response 4.83 s	Exploratory/descriptive support only; no claim of superiority without matched fixed-policy replay

## Data Availability

The original contributions presented in this study are included in the article. Further inquiries can be directed to the corresponding author.

## Abbreviations

This table standardizes navigation, estimation, integrity, and fault-management terminology. Throughout the manuscript, HMI denotes Hazardously Misleading Information, while an individual occurrence is written consistently as “HMI-event”. Human–machine interaction is written in full to avoid abbreviation ambiguity.

**Abbreviation**	**Definition**
AIS	Automatic Identification System
COG	Course over ground
ECE	Expected calibration error
EKF	Extended Kalman filter
FDI	Fault detection and isolation
GNSS	Global Navigation Satellite System
HMI	Hazardously misleading information
IMU	Inertial measurement unit
LiDAR	Light detection and ranging
PL	Protection level
RTK	Real-time kinematic
SOG	Speed over ground
